# Synthesis and vibrational spectroscopy of ^57^Fe-labeled models of [NiFe] hydrogenase: first direct observation of a nickel–iron interaction†
†Electronic supplementary information (ESI) available: Experimental procedures, spectral data, computational chemistry details, animated vibrational modes as GIFs. See DOI: 10.1039/c4cc04572f



**DOI:** 10.1039/c4cc04572f

**Published:** 2014-09-19

**Authors:** David Schilter, Vladimir Pelmenschikov, Hongxin Wang, Florian Meier, Leland B. Gee, Yoshitaka Yoda, Martin Kaupp, Thomas B. Rauchfuss, Stephen P. Cramer

**Affiliations:** a Department of Chemistry , University of Illinois at Urbana-Champaign , Urbana , IL 61801 , USA . Email: schilter@illinois.edu; b Institut für Chemie , Technische Universität Berlin , 10623 Berlin , Germany; c Department of Chemistry , University of California , Davis , CA 95616 , USA . Email: spjcramer@ucdavis.edu; d Physical Biosciences Division , Lawrence Berkeley National Laboratory , Berkeley , CA 94720 , USA; e JASRI , SPring-8 , Sayo-gun , Hyogo 679-5198 , Japan

## Abstract

A new route to iron carbonyls has enabled synthesis of ^57^Fe-labeled [NiFe] hydrogenase mimic (OC)_3_^57^Fe(pdt)Ni(dppe). Its study by nuclear resonance vibrational spectroscopy revealed Ni–^57^Fe vibrations, as confirmed by calculations. The modes are absent for [(OC)_3_^57^Fe(pdt)Ni(dppe)]^+^, which lacks Ni–^57^Fe bonding, underscoring the utility of the analyses in identifying metal–metal interactions.

## Abstract 2

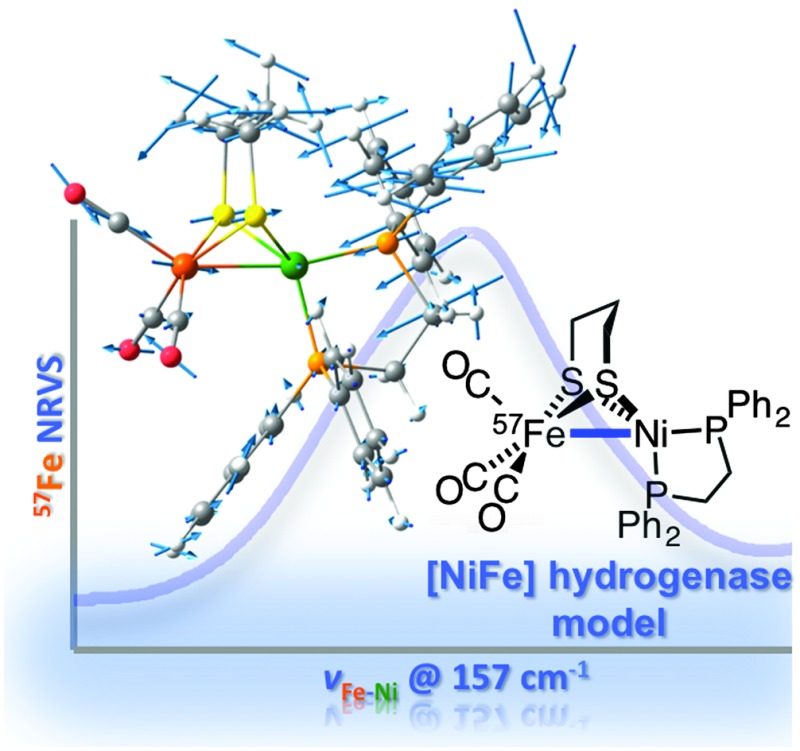
Isotopically labelled Ni^57^Fe models of the [NiFe] hydrogenase active site have been prepared and studied with nuclear resonant vibrational spectroscopy, enabling direct characterization of metal–metal bonding.

## 

Despite our extremely low atmospheric concentration of dihydrogen (∼1 ppm), this substrate is a key metabolite of many anaerobic bacteria.^1^ In such living systems can be found the most prevalent enzymes for hydrogen processing, the nickel–iron hydrogenases ([NiFe]–H_2_ases).^1,2^ These electrocatalysts specifically mediate the redox reaction H_2_ ⇌ 2H^+^ + 2e^–^ at several hundred turnovers per second.^3^ Their heterobimetallic active sites exist in several states, some of which are summarized below (Fig. 1, left and centre).

**Fig. 1:**
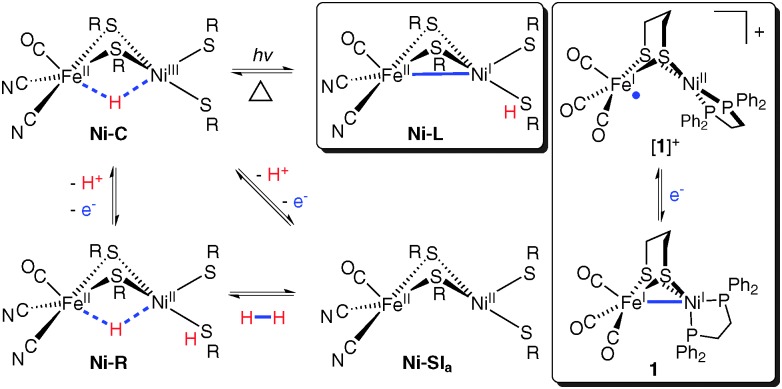
Key [NiFe]–H_2_ase states (left and centre) and two model complexes (right).

The active sites feature Ni bound to four cysteinato residues, two of which bridge to an Fe(CO)(CN)_2_ fragment. In the Ni–C state, Ni(iii)Fe(ii) centres bind a bridging hydride (H^–^), reductive elimination of which affords Ni–L.^4^ Thus, H^+^ is abstracted by a terminal *cys* ligand (Fig. 1, centre top), leaving a Ni(i)Fe(ii) core with a 2e^–^ bond between the metals.^4^

The use of vibrational spectroscopy to study [NiFe]–H_2_ase is convenient in that its active site features chromophores easily identifiable by such techniques. Spectral analyses are often aided by comparison to data from synthetic models^5^ whose structures are well understood. Specificity for ^57^Fe-coupled modes is afforded by nuclear resonance vibrational spectroscopy (NRVS, *vide infra*). This has recently enabled observation of characteristic Fe–CN/Fe–CO bending and stretching modes in [NiFe]–H_2_ase (Ni–A and Ni–R) and the Fe subsite model [Fe(benzenedithiolato)(CN)_2_CO]^2–^.^6^ However, no NRVS studies have reported on metal–metal bonding, which is expected for low-valent clusters like Ni–L and its models.^4,7^

A near-complete [NiFe]–H_2_ase mimic is the Ni(ii)Fe(i) species [(OC)_3_Fe(pdt)Ni(dppe)]^+^ ([**1**]^+^, pdt^2–^ = ^–^S(CH_2_)_3_S^–^; dppe = 1,2-bis(diphenylphosphino)ethane), a model for Ni–L, albeit with metal oxidation states reversed (Fig. 1, right).^8^ This *S* = 1/2 model is prepared from (OC)_3_Fe(pdt)Ni(dppe) (**1**),^9^ itself the subject of density functional theory (DFT) and resonance Raman investigations.^10^ Disclosed here is methodology for ^57^Fe-labeled prototypes [(OC)_3_^57^Fe(pdt)Ni(dppe)]^0/+^ ([**1**]^0/+^), enabling the study of metal–metal bonding with NRVS.

The Ni(i)Fe(i) complex **1** is usually accessed by interaction of (pdt)Ni(dppe) with an Fe carbonyl such as Fe_2_(CO)_9_ or Fe(CO)_4_I_2_.^11–14^ The precursor to these, Fe(CO)_5_, is not conveniently prepared from elemental Fe, a factor that necessitated a new route adaptable to ^57^Fe incorporation. Thus, metallic Fe was converted to the organo-soluble FeI_2_ source Fe_2_I_4_(^i^PrOH)_4_,^15^ which, upon combination with (pdt)Ni(dppe), gave the known diiodide I_2_Fe(pdt)Ni(dppe).^14^ While the diiodide does not bind CO in CH_2_Cl_2_, when treated with AgBF_4_ it converts to the putative electrophile ‘[IFe(pdt)Ni(dppe)]^+^’ (or perhaps its dimer), which undergoes carbonylation to afford [(OC)_3_FeI(pdt)Ni(dppe)]^+^ ([**1**I]^+^).^14^ Reduction with CoCp_2_ gave **1** in yields comparable to the (pdt)Ni(dppe)/Fe_2_(CO)_9_ route.^11^

In adapting the synthesis to the Ni^57^Fe target, elemental ^57^Fe was oxidized to ^57^Fe_2_I_4_(^i^PrOH)_4_,^12^ which was converted into violet [(OC)_3_^57^FeI(pdt)Ni(dppe)]^+^ (ESI-MS: *m*/*z* 829.5 [**1′**I]^+^ *vs.* 828.9 [**1**I]^+^), with reduction affording green (OC)_3_^57^Fe(pdt)Ni(dppe) (**1′**) in 35% yield (Scheme 1).

**Scheme 1:**
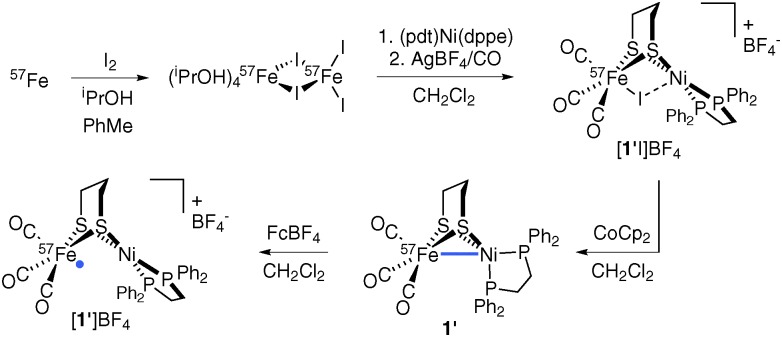


While *ν*_CO_ energies and analytical and ^31^P{^1^H} NMR data for **1′** are virtually identical to those of natural abundance **1**, LI-FDI-MS (liquid introduction field desorption ionization mass spectrometry) analyses are telling. Soft ionization of non-polar **1′** and **1** allowed for the detection of parent cations at *m*/*z* 702.9 and 701.9, respectively (Fig. S4, ESI†). Oxidation of **1′** with FcBF_4_ afforded mixed-valent salt [**1′**]BF_4_, whose EPR signal is broadened relative to that of [**1**]BF_4_ due to hyperfine interactions.

Investigations into Ni–Fe bonding in the new ^57^Fe-labeled variants of the reduced and oxidized complexes (respectively **1′** and [**1′**]^+^) were undertaken using NRVS. This technique, enabled by the development of third generation synchrotron sources, insertion devices and advanced X-ray optics,^16–18^ involves scanning an extremely monochromatic (*e.g.* 0.8 meV) X-ray beam through a nuclear resonance of a Mössbauer-active isotope (*e.g.* 14.4 keV for ^57^Fe). Subsequent relaxation causes the generation/annihilation of phonons, the detection of which reveals all modes in which the ^57^Fe nucleus moves along the direction of the incident X-ray. NRVS has several advantages over traditional IR and resonance Raman spectroscopies,^19^ not least in terms of element and isotope specificity and absence of the optical selection rules, which have allowed for the resolution of *ν*_Fe–X_ (X = S, P, Cl, CO, CN, NO) vibrations in complicated systems.^6,13,19,20^ This relatively new but powerful technique in inorganic and biological iron chemistry is applied here to [**1′**]^0/+^. The analysed spectra in terms of ^57^Fe partial vibrational density of states (PVDOS) are given in Fig. 2.

**Fig. 2:**
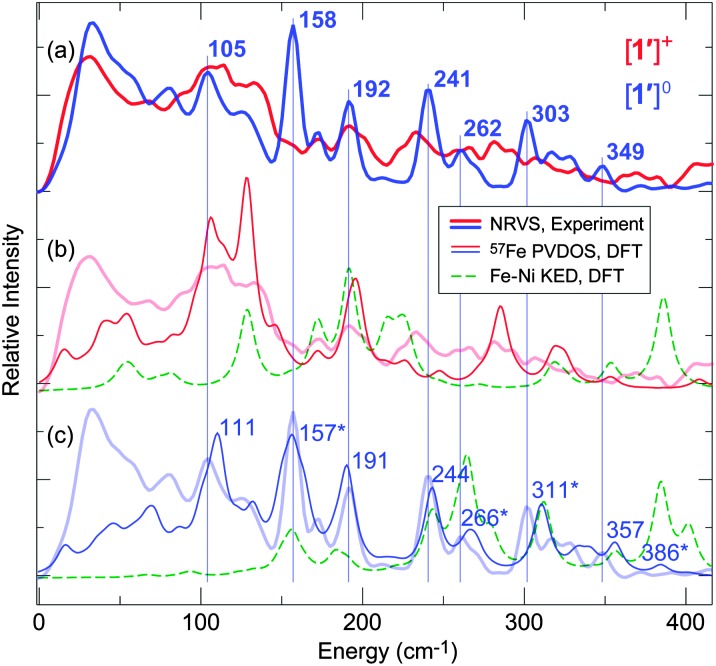
Observed NRVS spectra for [**1′**]^+^ (thick red lines, (a) and (b)) and **1′** (thick blue lines, (a) and (c)) *vs.* DFT calculated ^57^Fe PVDOS spectra for [**1′**]^+^ (thin red line, (b)) and **1′** (thin blue line, (c)). Calculated Fe–Ni KED (green) is given in (b) for [**1′**]^+^, and in (c) for **1′**. Key bands observed for **1′** are labelled in (a), with DFT counterparts in (c) indicated by vertical lines. Modes giving rise to bands with significant Fe–Ni character are marked (*) and shown in Fig. 3 and Fig. S10 (ESI†). For 0–650 cm^–1^ spectra, see Fig. S9 (ESI†).

Intense bands assigned to *ν*_Fe–CO_ and *δ*_Fe–CO_ modes were observed at 440–630 cm^–1^, with full-range NRVS spectra and Fe–C(O) kinetic energy distribution (KED) diagrams presented in Fig. S9 (ESI†). Similar signals were found for [NiFe]–H_2_ase.^6^ It was expected that *ν*_Fe–S_, *δ*_Fe–S_ and *ν*_Fe–Ni_ bands, if observable, would lie at low energies (≤400 cm^–1^). Upon comparing data for **1′** and [**1′**]^+^ (Fig. 2a), a sharp and prominent NRVS peak at 158 cm^–1^ for **1′** was noticed. This band, absent from the spectra of [**1′**]^+^, was tentatively ascribed to vibration of the Ni–Fe bond, such interactions not being significant in [**1′**]^+^. Differences in NRVS data of **1′** and [**1′**]^+^ were less marked in other spectral regions, although peaks for [**1′**]^+^ were broader.

The assignment of vibrational bands was elaborated using DFT calculations on [**1′**]^0/+^ as detailed in the ESI;† simulated NRVS (^57^Fe PVDOS) and Fe–Ni KED diagrams for [**1′**]^+^ and **1′** are also presented in Fig. 2b and c, respectively. Band positions and intensities in the calculated and observed spectra are largely in agreement (particularly in Fig. 2c; see also Fig. S9, ESI†). In the case of [**1′**]^+^, some differences are assigned to impurities (**1′** and/or [**1′**H]^+^). The band for **1′** at 158 cm^–1^, calculated by DFT at 157 cm^–1^, indeed involves stretching of the Fe–Ni bond symmetrical to a Ni–P1 stretch (see Fig. 3, and the ESI† for animations). Such a vibrational coupling in the Fe–Ni–P1 triad implicates a strong Fe–Ni interaction, and it has no complement in the normal modes pool calculated for [**1**]^+^.

**Fig. 3:**
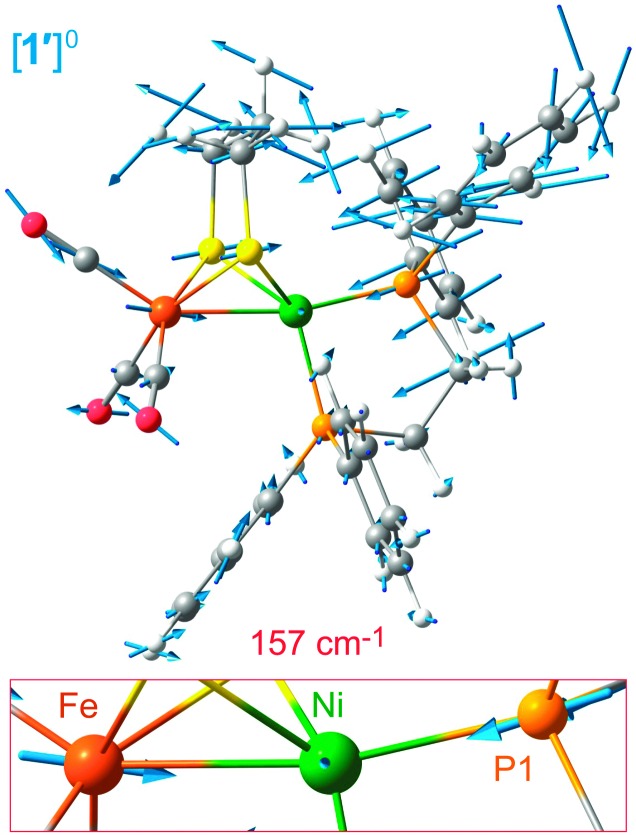
Scaled arrow depiction of nuclear displacements for the normal mode calculated for **1′** at 157 cm^–1^ (a symmetric Fe–Ni–P1 stretch, see corresponding ^57^Fe PVDOS band in Fig. 2c). Key [**1′**]^0/+^ modes are animated in the ESI.†

While very prominent in NRVS (owing to the large ^57^Fe displacement), the 157 cm^–1^ band is weaker (∼5%) in the Ni–Fe KED diagram, which reflects *relative* motion of Fe and Ni. Other Ni–Fe stretches, such as those calculated at 266, 311, and 386 cm^–1^ (see in Fig. 2c), are considerably stronger (representing 13%, 9%, and 11% total Ni–Fe KED, respectively). Yet only the first two modes can be associated with bands observed at 262 and 303 cm^–1^, while the last one has vanishing NRVS intensity (Fig. 2a). The modes at 266, 311, and 386 cm^–1^ have lower ^57^Fe PVDOS intensity as they involve displacement mostly of Ni (rather than Fe), this movement being evident from the DFT results (Fig. S10, ESI†). Analysis of Fe–Ni vibrations is further complicated by vibrational coupling to C/P/S atoms, in particular the bridging S donors. Notably, mixed *ν*_Fe–Ni_ modes in the 220–360 cm^–1^ region involving up to 14% contribution from the Fe–Ni stretch were also reported for **1**.^10^

Structural (X-ray diffraction) and DFT studies on unlabeled [**1**]^0/+^ have suggested that the reduced Ni(i)Fe(i) species may feature Ni–Fe bonding,^9^ while the oxidized Ni(ii)Fe(i) does not.^8^ This was confirmed by re-analysing bonding in [**1**]^0/+^ using ELF,^21^ ELI-D,^22^ and QTAIM^23^ electron density-based methods (Fig. 4; Fig. S11 and S12, ESI†), all of which indicated a Ni–Fe bond in **1**. Notably, the ELF/ELI-D bond attractor and the bond critical point found by QTAIM are shifted from the Ni–Fe vector away from the bridging S atoms, such that Fe(i) is (pseudo)octahedral. Bonding involves overlap of two singly-occupied d(*z* ^2^) orbitals and is absent when Ni is oxidized, with [**1**]^+^ featuring a Fe-localized d(*z* ^2^) SOMO. In contrast, Ni–L has a SOMO with Ni d(*z* ^2^) and d(*x* ^2^ – *y* ^2^) character,^4^ and a 2e^–^ Ni→Fe dative bond (optimized Fe–Ni distance: 2.47 Å).^4^ While [**1**]^+^ and Ni–L have the same electron count for the [NiFe]^3+^ site, only one has Ni–Fe bonding. A key distinction is Ni geometry, which is planar in [**1**]^+^ but SF_4_-like in Ni–L.

**Fig. 4:**
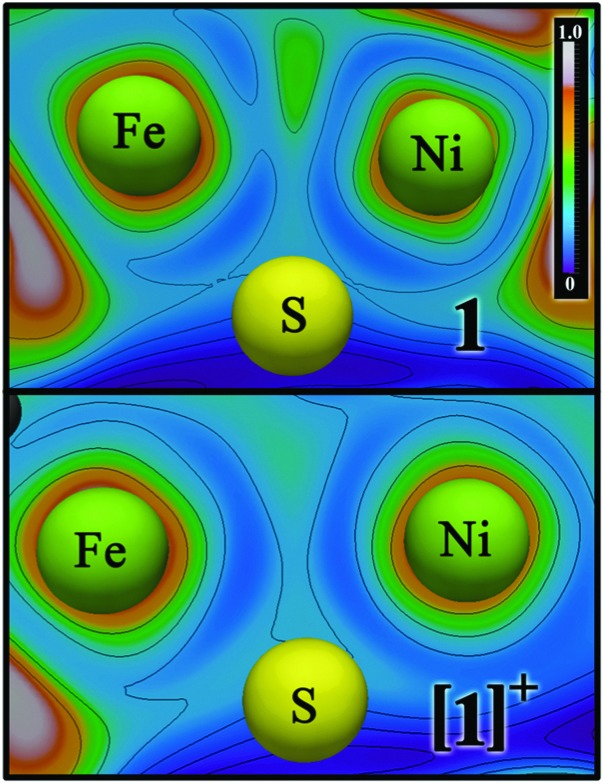
Electron localization function (ELF) analysis of the Ni–Fe bonding in [**1**]^0/+^ (top/bottom). Ni–Fe bond attractor position for [**1**]^0^ is indicated by the localized area in green (center-top), absent for [**1**]^+^. See Fig. S11 and S12 (ESI†) for alternative bonding representations.

Since the Fe(i) centre in [**1**]^+^ is electron poor (average *ν*_CO_ = 2010 cm^–1^)^8^ it cannot supply 2e^–^ for a Fe→Ni coordinate bond. Another possibility, a covalent Ni–Fe bond, is unlikely due to the low donicity of Ni(ii). The metal centres in [**1**]^+^ are distant (our DFT result: 2.80 Å), while those in electron-rich **1** (average *ν*_CO_ = 1977 cm^–1^) are sufficiently proximal (experimental: 2.47 Å,^9^ DFT: 2.46 Å) for covalent Fe–Ni bonding (see Table S2 and Fig. S3, ESI† for structural and IR details).

Obtaining direct evidence of metal–metal bonding in molecular systems is nontrivial, and distances do not guarantee presence/absence of bonding. For example, EXAFS studies reveal similar Fe–Ni distances for Ni–L and Ni–C,^24^ despite Ni not being bonded to Fe in the latter. This highlights that (i) [NiFe]–H_2_ase active site ligation is inflexible (relative to [**1**]^0/+^) and that (ii) EXAFS/XRD studies typically only afford nuclear positions *via* core electron density, with bonding electron density between metals being poorly resolved.^25^ In rare cases when very high quality single crystal data are obtained, multipole analysis, followed by topological analysis of static electron density (*versus* inspection of difference maps) can give insights into metal–metal bonding, as exemplified by (re)investigation of the archetypal Mn_2_(CO)_10_.^26^

Metal–metal bonding is common in organoiron chemistry, but it also plays a role in the reduced states of some metalloenzymes, stabilizing low-valent metal centres poised for substrate activation.^7^ In addition to [NiFe]–H_2_ase, Ni–Fe interactions are also proposed for carbon monoxide dehydrogenase (CODH),^7,27^ with Ni–Ni and Fe–Fe bonds being present in acetyl-CoA synthetase (ACS)^7^ and [FeFe]–H_2_ase,^28^ respectively. NRVS is demonstrably effective in unambiguous identification of low energy ^57^Fe-coupled modes.^13,20,29^ Compared to IR and resonance Raman, it avoids interference from solvent and ‘fingerprint’ bands, enabling identification of Fe-coupled vibrations, such as the 158 cm^–1^ *ν*_Fe–Ni_ mode here. The use of NRVS to probe iron–metal modes in everything from small molecules to iron enzymes is anticipated to provide a wealth of information on these catalysts.

While primordial routes to iron carbonyls have been reported (*e.g.* the one-pot preparation of (CO)_3_Fe(pdt)Fe(CO)_3_ from FeCl_2_),^30^ they are typically limited in scope and reproducibility. In contrast, the Fe_2_I_4_(^i^PrOH)_4_/CO/CoCp_2_ strategy will likely be generalizable and afford iron carbonyls of relevance to H_2_ases and organoiron chemistry in general. With the isolation of ^57^Fe-labeled [NiFe]–H_2_ase mimics [**1′**]^0/+^, the element and isotope selectivity of NRVS was exploited to obtain the first vibrational spectroscopic evidence of Fe–Ni interactions. These results serve as an important benchmark, opening the door to work probing metal–metal bonding in redox-active H_2_ase enzymes as well as related enzymatic and model systems.

Thanks are given to Drs Mark J. Nilges and Haijun Yao for assistance with EPR and LI-FDI-MS, respectively. Financial support was provided by the National Institutes of Health (GM061153-10 to T.B.R. and GM-65440 to S.P.C.), U.S. Department of Energy Office of Biological and Environmental Research (DOE OBER) (S.P.C.), and the ‘Unifying Concepts in Catalysis' initiative of the German Research Council (V.P., F.M., and M.K.). NRVS experiments performed at SPring-8 BL09XU were funded by JASRI (beamtime proposal 2013A0032).

## References

[cit1] Vignais P. M., Billoud B. (2007). Chem. Rev..

[cit2] Ogata H., Lubitz W., Higuchi Y. (2009). Dalton Trans..

[cit3] Frey M. (2002). ChemBioChem.

[cit4] Kampa M., Pandelia M.-E., Lubitz W., van Gastel M., Neese F. (2013). J. Am. Chem. Soc..

[cit5] Kaur-Ghumaan S., Stein M. (2014). Dalton Trans..

[cit6] Kamali S., Wang H., Mitra D., Ogata H., Lubitz W., Manor B. C., Rauchfuss T. B., Byrne D., Bonnefoy V., Jenney F. E., Adams M. W. W., Yoda Y., Alp E., Zhao J., Cramer S. P. (2013). Angew. Chem., Int. Ed..

[cit7] Lindahl P. A. (2012). J. Inorg. Biochem..

[cit8] Schilter D., Nilges M. J., Chakrabarti M., Lindahl P. A., Rauchfuss T. B., Stein M. (2012). Inorg. Chem..

[cit9] Zhu W., Marr W. A. C., Wang Q., Neese F., Spencer D. J. E., Blake A. J., Cooke P. A., Wilson C., Schröder M., Huynh M. T., Schilter D., Hammes-Schiffer S., Rauchfuss T. B. (2005). Proc. Natl. Acad. Sci. U. S. A..

[cit10] Shafaat H. S., Weber K., Petrenko T., Neese F., Lubitz W. (2012). Inorg. Chem..

[cit11] Barton B. E., Rauchfuss T. B. (2010). J. Am. Chem. Soc..

[cit12] Carroll M. E., Chen J., Gray D. E., Lansing J. C., Rauchfuss T. B., Schilter D., Volkers P. I., Wilson S. R. (2014). Organometallics.

[cit13] Guo Y., Wang H., Xiao Y., Vogt S., Thauer R. K., Shima S., Volkers P. I., Rauchfuss T. B., Pelmenschikov V., Case D. A., Alp E., Sturhahn W., Yoda Y., Cramer S. P. (2008). Inorg. Chem..

[cit14] Schilter D., Rauchfuss T. B. (2012). Dalton Trans..

[cit15] Nunes G. G., Bottini R. C. R., Reis D. M., Camargo P. H. C., Evans D. J., Hitchcock P. B., Leigh G. J., Sá E. L., Soares J. F. (2004). Inorg. Chim. Acta.

[cit16] Seto M., Yoda Y., Kikuta S., Zhang X. W., Ando M. (1995). Phys. Rev. Lett..

[cit17] Sturhahn W., Toellner T. S., Alp E. E., Zhang X., Ando M., Yoda Y., Kikuta S., Seto M., Kimball C. W., Dabrowski B. (1995). Phys. Rev. Lett..

[cit18] Yoda Y., Imai Y., Kobayashi H., Goto S., Takeshita K., Seto M. (2012). Hyperfine Interact..

[cit19] Smith M. C., Xiao Y., Wang H., George S. J., Coucouvanis D., Koutmos M., Sturhahn W., Alp E. E., Zhao J., Cramer S. P. (2005). Inorg. Chem..

[cit20] Tonzetich Z. J., Wang H., Mitra D., Tinberg C. E., Do L. H., Jenney, Jr. F. E., Adams M. W. W., Cramer S. P., Lippard S. J. (2010). J. Am. Chem. Soc..

[cit21] Becke A. D., Edgecombe K. E., Savin A., Jepsen O., Flad J., Andersen O. K., Preuss H., Vonschnering H. G., Kohout M., Savin A. (1990). J. Chem. Phys..

[cit22] Kohout M., Kohout M., Pernal K., Wagner F. R., Grin Y. (2004). Int. J. Quantum Chem..

[cit23] Bader R. F. W., Atoms in Molecules: A Quantum Theory, Oxford University Press, Oxford, 1990.

[cit24] Whitehead J. P., Gurbiel R. J., Bagyinka C., Hoffman B. M., Maroney M. J. (1993). J. Am. Chem. Soc..

[cit25] Koritsanszky T. S., Coppens P. (2001). Chem. Rev..

[cit26] Bianchi R., Gervasio G., Marabello D. (2000). Inorg. Chem..

[cit27] Jeoung J.-H., Dobbek H. (2009). J. Am. Chem. Soc..

[cit28] Nicolet Y., de Lacey A. L., Vernède X., Fernandez V. M., Hatchikian E. C., Fontecilla-Camps J. C. (2001). J. Am. Chem. Soc..

[cit29] Cramer S. P., Xiao Y., Wang H., Guo Y., Smith M. C. (2006). Hyperfine Interact..

[cit30] Volkers P. I., Boyke C. A., Chen J., Rauchfuss T. B., Whaley C. M., Wilson S. R., Yao H. (2008). Inorg. Chem..

