# Supplementary material for: Synthesis and vibrational spectroscopy of 57Fe-labeled models of [NiFe] hydrogenase: first direct observation of a nickel–iron interaction
Source: Chem Commun (Camb). 2014 Sep 19;50(88):13469–72. doi: 10.1039/c4cc04572f (PMC4191989; doi:10.1039/c4cc04572f)
Supplement: Supplementary file 1 [file CC-050-C4CC04572F-s001.pdf]

Electronic Supplementary Material (ESI) for Chemical Communications.
This journal is © The Royal Society of Chemistry 2014

Supplementary Information for:

Synthesis and Vibrational Spectroscopy of ^{57}Fe-Labeled Models of [NiFe] Hydrogenase: First Direct Observation of a Nickel-Iron Interaction

David Schilter,[a] Vladimir Pelmenschikov,[b] Hongxin Wang,[c,d] Florian Meier,[b] Leland B. Gee,[c] Yoshitaki Yoda,[e] Martin Kaupp,[b] Thomas B. Rauchfuss,[a] and Stephen P. Cramer[c,d]

[a]Department of Chemistry, University of Illinois at Urbana-Champaign, Urbana, IL 61801, USA
[b]Institut für Chemie, Technische Universität Berlin, 10623 Berlin, Germany
[c]Department of Chemistry, University of California, Davis, CA 95616, USA
[d]Physical Biosciences Division, Lawrence Berkeley National Laboratory, Berkeley, CA 94720, USA
[e]JASRI, SPring-8, Sayo-gun, Hyogo 679-5198, Japan

Contents

List of Figures 1
List of Tables 2
Syntheses 2
General considerations 2
Conversion of $I_2Fe(pdt)Ni(dppe)$ to $(OC)_3Fe(pdt)Ni(dppe)$ (**1**) 2
$(OC)_3{}^{57}Fe(pdt)Ni(dppe)$ (**1′**) 4
$[(OC)_3{}^{57}Fe(pdt)Ni(dppe)]BF_4$ ([**1′**]BF_4) 6
NRVS 7
DFT Calculations 8
References 14

List of Figures

Fig. S1 Positive ion ESI mass spectrum of [**1′**I]BF_4. 3
Fig. S2 $^{31}P\{^1H\}$ NMR spectrum (CH_2Cl_2, 202 MHz) of **1′** 4
Fig. S3 Observed DFT calculated IR spectra of [**1′**]$^+$ and **1′** in the ν_{CO} region. 5
Fig. S4 Positive ion LI-FDI mass spectrum of **1** and **1′** 5
Fig. S5 Positive ion ESI mass spectrum of [**1′**]BF_4. 6
Fig. S6 X-band EPR spectrum of [**1**]BF_4 and [**1′**]BF_4. 6
Fig. S7 Experimental and simulated X-band EPR spectrum of [**1′**]BF_4 7
Fig. S8 X-ray structures of **1** and [**1**]$^+$ analog $[(CO)_3Fe(pdt)Ni(dcpe)]^+$ 9
Fig. S9 Observed and DFT calculated NRVS (^{57}Fe PVDOS) data for **1′** and [**1′**]$^+$ 10
Fig. S10 Scaled arrow depiction of nuclear displacements for selected normal modes with significant Fe-Ni character calculated for **1′** (157, 266, 311, and 386 cm^{-1}) 11
Fig. S11 ELF and ELI-D analyses of the Fe-Ni bonding in [**1**]$^{0/+}$ 12
Fig. S12 QTAIM topologic and total electron density Laplacian analyses of the Fe-Ni bonding in [**1**]$^{0/+}$ 13

List of Tables

Table S1 EPR simulation parameters for the two conformers of [**1′**]BF_4 7
Table S2 Metal-metal and metal-ligand internuclear distances (Å) in [**1**]$^{0/+}$ from X-ray data analyses and DFT optimization 9
Table S3 Peak frequencies (cm^{-1}) for experimental NRVS/IR bands of [**1**]$^{0/+}$ and their equivalents from DFT calculations 10

Syntheses

General considerations

All reactions not involving CO were conducted in an MBraun glovebox equipped with a solvent purification system; the concentrations of O_2 and H_2O in the N_2 atmosphere were typically no higher than 2 and 0.2 ppm, respectively. Unless otherwise stated, all chemicals were purchased from commercial sources and used as received. The solvents CD_2Cl_2 and $^{i}PrOH$ were distilled from CaH_2 and $FcBF_4$ crystallized from CH_2Cl_2/hexanes. The complexes $Fe_2I_4(^{i}PrOH)_4$,[1] $^{57}Fe_2I_4(^{i}PrOH)_4$,[2] (pdt)Ni(dppe),[3] I_2Fe(pdt)Ni(dppe)[4] and $(OC)_3Fe$(pdt)Ni(dppe)[5] were prepared according to the literature methods. Chromatography was performed using SiO_2 (40 - 63 μm, 230 - 400 mesh) as the stationary phase. Solution IR spectra were recorded on a Perkin-Elmer Spectrum 100 FTIR spectrometer. LI-FDI- and ESI-MS data were collected using a Waters GCT Premier and Waters Micromass Quattro II spectrometer, respectively. In each case analytes were injected as dilute CH_2Cl_2 solutions. Analytical data were acquired using an Exeter Analytical CE-440 elemental analyzer. NMR data were acquired at room temperature, with samples under an atmosphere of N_2. ^{1}H, $^{13}C\{^{1}H\}$ and $^{31}P\{^{1}H\}$ NMR spectra were recorded on a Varian VXR500 spectrometer at 500, 126 and 202 MHz, respectively. Chemical shifts (in ppm) are referenced to $CHDCl_2/CH_2Cl_2$ (5.32 ppm for ^{1}H) and external 85% H_3PO_4 (0 ppm for ^{31}P). EPR spectra (~1 mM in CH_2Cl_2/PhMe, 1:1) were recorded on a Varian E-line 12″ Century Series X-band CW spectrometer at 110 K.

*Conversion of I_2Fe(pdt)Ni(dppe) to $(OC)_3Fe$(pdt)Ni(dppe) (**1**)*

A solution of I_2Fe(pdt)Ni(dppe) (26.2 mg, 30 μmol) in CH_2Cl_2 (1 mL) was treated with a suspension of $AgBF_4$ (5.8 mg, 30 μmol) in CH_2Cl_2 (2 mL) and the mixture stirred under CO (1 atm) in the absence of light. After 24 h, the suspension was filtered through Celite and the filtrate cooled to –28°C, whereupon $CoCp_2$ (11.3 mg, 60 μmol) in CH_2Cl_2 (1 mL) was added. The solution was concentrated to ~1 mL and chromatographed (~5 cm SiO_2, CH_2Cl_2 eluent). The first band, deep green in color, was collected and treated with CH_3CN (5 mL). The solution was concentrated to ~0.5 mL, whereupon a solid formed. The material was isolated by filtration, washed with CH_3CN (2 × 5 mL), Et_2O (2 × 5 mL) and pentane (2 × 5 mL), and dried briefly to afford the title compound as dark green crystals (13.9 mg, 20 μmol, 66%). Characterization data were identical to those previously reported.

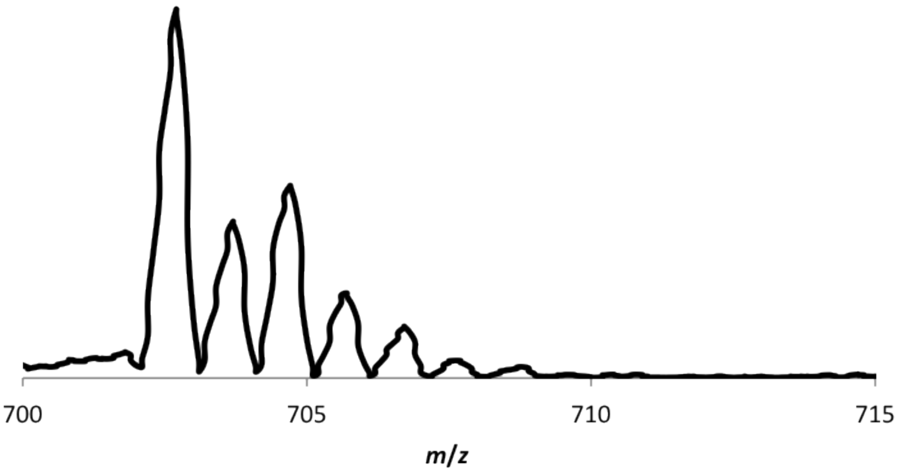

Fig. S1 Positive ion ESI mass spectrum of [**1′**I]BF_4.

*(OC)$_3$^{57}Fe(pdt)Ni(dppe) (**1'**)*

A suspension of $^{57}Fe_2I_4(^iPrOH)_4$ (34.5 mg, 40 μmol) in CH_2Cl_2 (1 mL) was treated with (pdt)Ni(dppe) (22.5 mg, 40 μmol) in CH_2Cl_2 (1 mL), and the mixture rapidly stirred for 2 h. A suspension of $AgBF_4$ (23.4 mg, 120 μmol) in CH_2Cl_2 (5 mL) was added and the mixture stirred under CO (1 atm) in the absence of light. After 24 h, the suspension was filtered through Celite and the filtrate cooled to –28°C, whereupon $CoCp_2$ (22.7 mg, 120 μmol) in CH_2Cl_2 (1 mL) was added. The solution was concentrated to ~1 mL and chromatographed rapidly (~5 cm SiO_2, CH_2Cl_2 eluent). The first band, deep green in color, was collected and treated with CH_3CN (5 mL). The solution was concentrated to ~0.5 mL, whereupon a solid formed. The material was isolated by filtration, washed with CH_3CN (2 × 5 mL), Et_2O (2 × 5 mL) and pentane (2 × 5 mL), and dried briefly to afford the title compound as dark green crystals (9.9 mg, 14 μmol, 35%).
$^{31}P\{^1H\}$ NMR (CH_2Cl_2) 61.8 ppm. FTIR (CH_2Cl_2): ν_{CO} = 2029, 1957 cm^{-1}. LI-FDI-MS: *m/z* 702.9 $[M]^+$, 674.9 $[M - CO]^+$. Anal. calcd for $C_{32}H_{30}O_3S_2P_2Ni^{57}Fe$: C, 54.57; H, 4.29; N, 0.00. Found: C, 54.26; H, 3.86; N, 0.00.

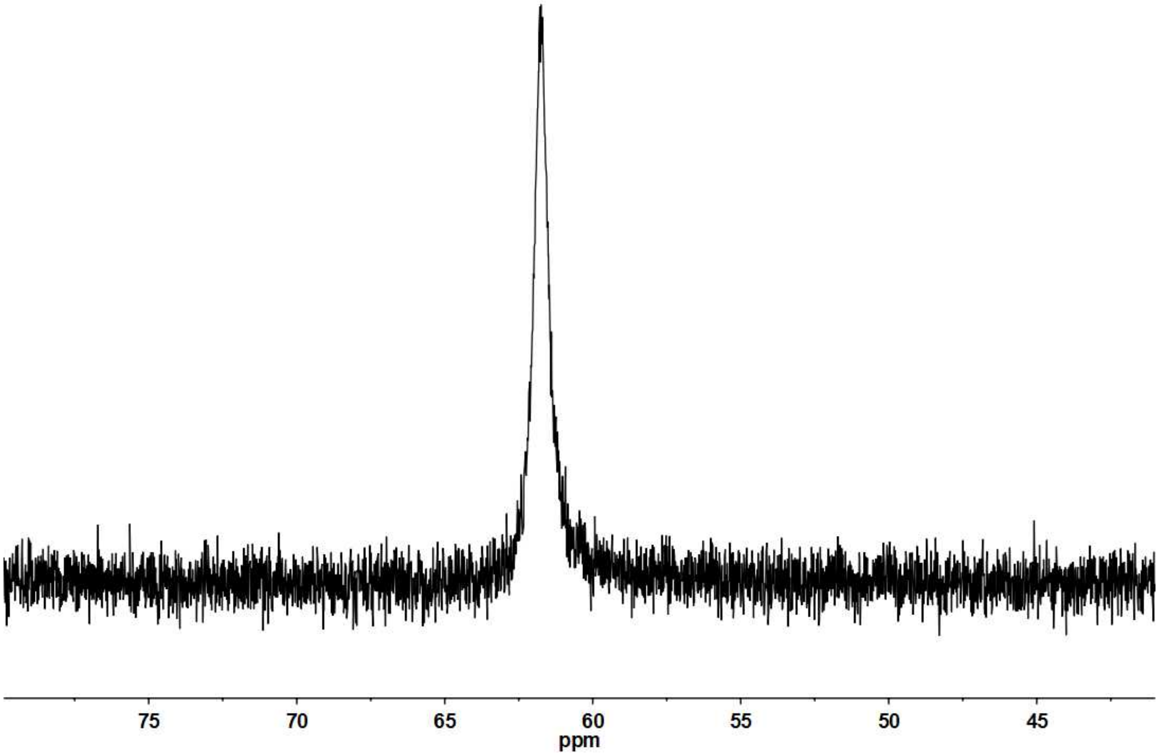

Fig. S2 $^{31}P\{^1H\}$ NMR spectrum (CH_2Cl_2, 202 MHz) of **1′**.

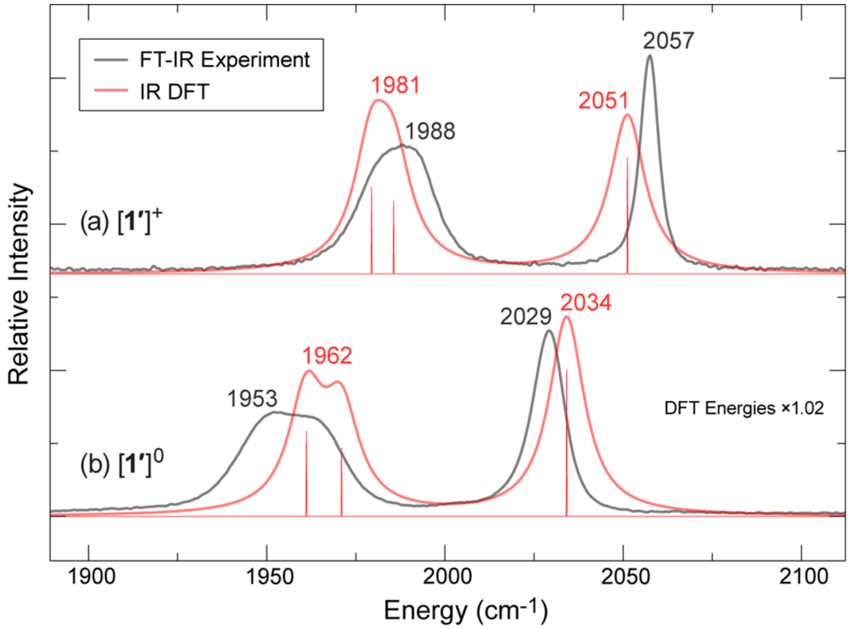

Fig. S3 Observed (black) and DFT calculated (red) IR spectra of (a) [**1′**]$^+$ and (b) **1′** in the ν_{CO} region. The stick spectra from DFT indicate three ν_{CO} normal modes positions, which are mixed 3×C–O stretches (two asymmetric stretches forming the lower frequency band, and one symmetric stretch for the higher frequency band; see the electronic SI for the mode animations). CH_2Cl_2 solvent was used for observed spectra. 102% scaling of DFT vibrational frequencies was applied.

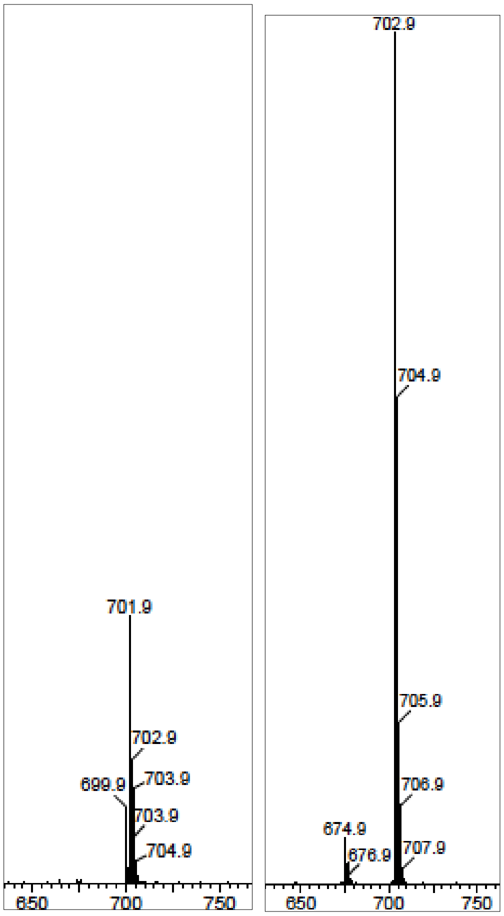

Fig. S4 Positive ion LI-FDI mass spectrum of **1** (left) and **1′** (right).

*[(OC)$_3$^{57}Fe(pdt)Ni(dppe)]BF$_4$ ([**1′**]BF$_4$)*

A stirred solution of **1′** (10.6 mg, 15 μmol) in CH_2Cl_2 (1 mL) was treated with $FcBF_4$ (4.1 mg, 15 μmol) in CH_2Cl_2 (1 mL). After 1 min, pentane (20 mL) was added and the mixture allowed to stand overnight at −28°C. The solids were isolated by filtration, washed with pentane (2 × 1 mL), and dried briefly to afford the title compound as a brown-olive powder (5.0 mg, 6.3 μmol, 42%).
FTIR (CH_2Cl_2): ν_{CO} = 2057, 1988 cm^{-1}. ESI-MS: *m/z* 702.7 [M – BF_4^-]$^+$. Anal. calcd for $C_{32}H_{30}O_3S_2P_2Ni^{57}FeBF_4$: C, 48.59; H, 3.82; N, 0.00. Found: C, 48.18; H, 3.89; N, 0.35.

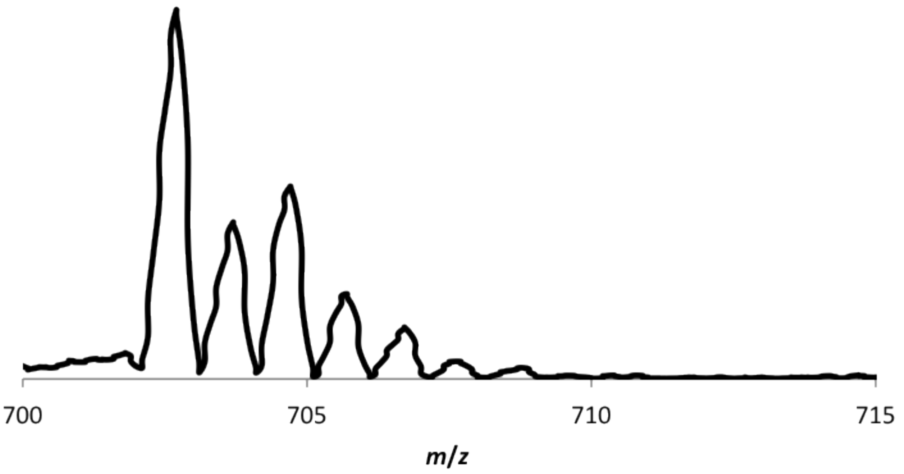

Fig. S5 Positive ion ESI mass spectrum of [**1′**]BF_4.

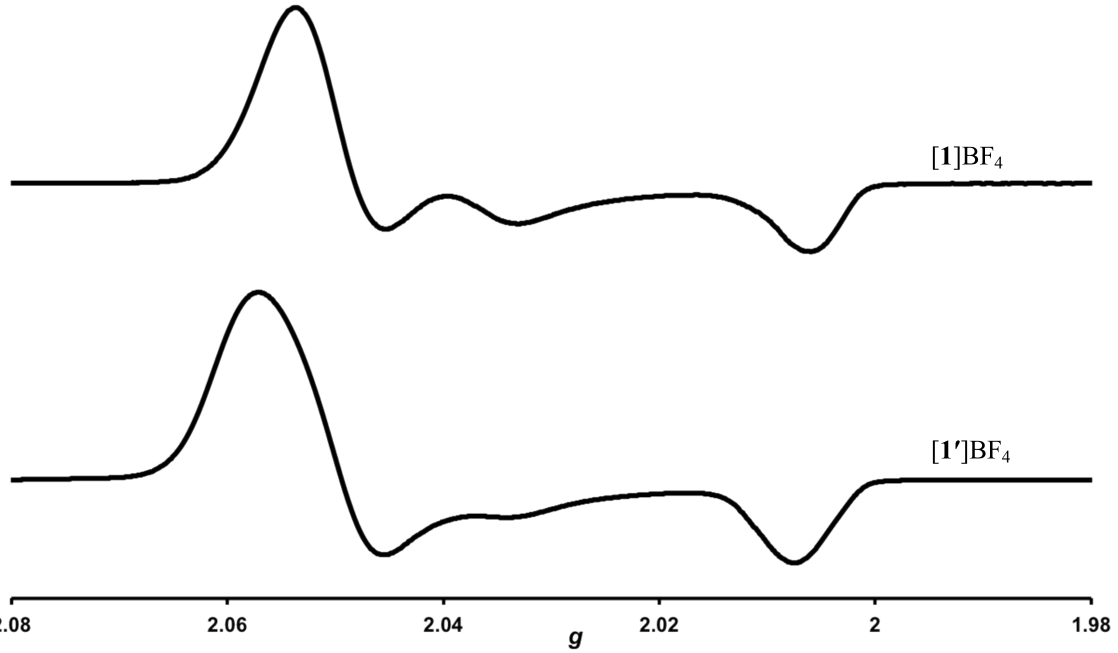

Fig. S6 X-band EPR spectrum of [**1**]BF_4 and [**1′**]BF_4.

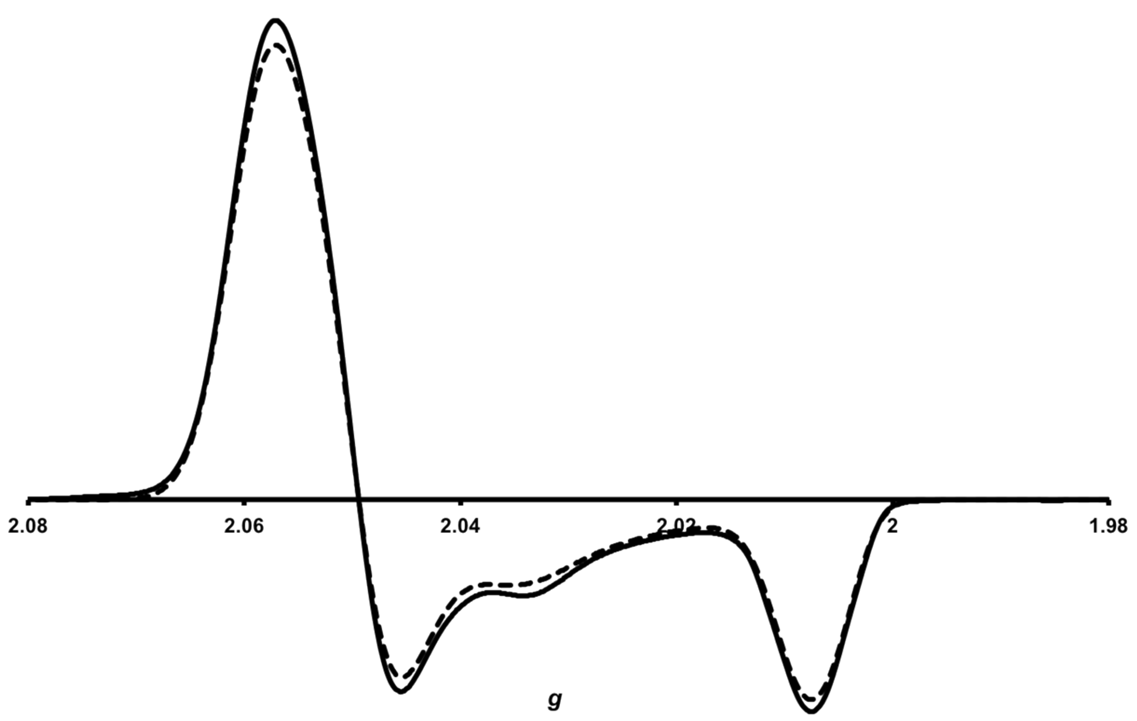

Fig. S7 X-band EPR spectrum of [**1′**]BF_4 (solid trace) and its simulated spectrum (dashed trace).

Table S1 EPR simulation parameters for [**1′**]BF_4. The species is present as two conformers, related by a ring flip in the Fe(pdt) chelate ring.

***g*-factor**	***A*(^{57}Fe) /MHz**	**line width /G**	**relative abundance**
2.054, 2.053, 2.007	24, 27, 14	9, 14, 8	0.84
2.055, 2.038, 2.009	49, 38, 27	16, 12, 10	0.16

NRVS

Data were recorded according to a published procedure[6,7] at SPring-8 BL09XU in Japan. A high heat load [Si(1,1,1)×Si(1,1,1)] monochromator (HHLM) produced 14.4 keV radiation with ~1.0 eV resolution, and a high energy resolution monochromator (HRM) [Ge(4,2,2)×2Si(9,7,5)] produced 14.4 keV radiation with 0.8 meV resolution. The beam flux was ~ 1.4×10^9 photons/s. The solids **1′** and [**1′**]BF_4 were mounted using 1-propanol as the medium, measurements being taken at 30-50 K using a liquid helium cryostat. Sample temperatures were calculated using the ratio of anti-stokes/stokes intensities according to: $\frac{S(-E)}{S(E)} = e^{-\frac{E}{kT}}$.[8-10] Delayed nuclear fluorescence and Fe K fluorescence (from internal conversion) were recorded with a 2×2 APD array. NRVS data were recorded with a step size of 0.28 meV, the counting time per point being 5 s. Analysis was performed using the PHOENIX software package.[7,9]

DFT Calculations

The initial coordinates for the DFT calculations on the $[(OC)_3{}^{57}Fe(pdt)Ni(dppe)]^{0/+}$ ($[\mathbf{1'}]^{0/+}$) model compounds were based on the X-ray structures of the natural abundance complexes **1**[11] and $[(OC)_3Fe(pdt)Ni(dcpe)]^+$,[12] respectively (Fig. S8). The latter Ni(II)Fe(I) complex was used as the structural reference for $[\mathbf{1'}]^+$, subject to dcpe to dppe ligand replacement. The structure optimization and subsequent normal mode analysis were done using GAUSSIAN 09,[13] based on the densities exported from single point calculations using JAGUAR 7.9.[14] The BP86[15,16] functional and the LACV3P** basis set as implemented in JAGUAR 7.9 were employed. For the first- and second-row elements, LACV3P** implies 6-311G** triple-zeta basis sets including polarization functions. For the Fe and Ni atoms, LACV3P** consists of a triple-zeta quality basis set for the outermost core and valence orbitals, and the quasirelativistic Los Alamos effective core potential (ECP) for the innermost electrons. The $[\mathbf{1'}]^{0/+}$ species environment was considered via self-consistent reaction field (SCRF) polarizable continuum model using the integral equation formalism (IEF-PCM)[17] as implemented in GAUSSIAN 09 with the IEF-PCM parameters at their default values for water. Based on the normal mode outputs, ^{57}Fe partial vibrational density of states (PVDOS) spectra which complement the NRVS experiment were generated using the Q-SPECTOR program. Q-SPECTOR is an in-house Python tool for analysis of the normal modes calculated using computational chemistry software (here, GAUSSIAN 09), successfully applied earlier.[18-21] To account for the resolution of the present NRVS and IR experiments, the simulated spectra were broadened by convolution with a 6 cm^{-1} Lorentzian. A homogeneous empirical scaling of the calculated frequencies was applied to provide improved (and otherwise apparent) mapping between the observed and calculated spectral features: by 108/98% in the 0-400/400-650 cm^{-1} ranges, respectively, for the NRVS bands (Fig. S9), and by 102% for the ν_{CO} IR bands around ~2000 cm^{-1} (Fig. S3). The observed vs. calculated bands correspondence is further provided in Table S3.

Our results on the electronic structure of ^{57}Fe $[\mathbf{1'}]^{0/+}$ complexes are in line with previous DFT studies on the natural abundance variants $[\mathbf{1}]^0$ [11,22] and $[\mathbf{1}]^+$.[12] The reduced $[\mathbf{1'}]^0$ species (metal oxidation levels Ni(I)Fe(I), total spin $S = 0$) displays essentially zero spin population at both the metal centers even in case open-shell (spin-unrestricted) DFT formalism is applied. The calculations on the $1e^-$ oxidized $[\mathbf{1'}]^+$ species (metal oxidation levels Ni(II)Fe(I), total spin $S = ½$) verify the unpaired electron to reside at the Fe(I) center. Notably, when $1e^-$ was added to the $[\mathbf{1'}]^+$ species solution (*i.e.*, using the optimized $[\mathbf{1'}]^+$ structure *and* electron density as an initial guess) and open-shell $S = 0$ singlet formalism was applied, the resulting species displayed Ni(I)Fe(I) character and zero spin populations, similarly to $[\mathbf{1'}]^0$.

Notably, our attempts to vary the DFT methodology described above using (*i*) alternative functionals (non-hybrid PBE[23,24] and hybrid B3LYP[25,26]), (*ii*) larger basis set (adding '+' diffuse functions), (*iii*) two-body D3 dispersion corrections by Grimme *et al.*,[27,28] and (*iv*) options on the SCRF procedure did not produce any better correspondence between the calculated ^{57}Fe PVDOS and experimental NRVS spectra for $[\mathbf{1'}]^{0/+}$.

The wavefunctions were analyzed using the DGrid program[29] by means of the electron localization function (ELF),[30] the electron localizability indicator based on the parallel-spin electron pair density (ELI-D)[31] and the Laplacian of the electron density and bond-path analyses within the quantum theory of atoms in molecules (QTAIM).[32] For this purpose, the Kohn-Sham orbitals of the single point calculations in GAUSSIAN 09 (basis sets and functional see above) were transferred to DGrid and the examined property was calculated on a grid with 50 points per Bohr. The results of ELF, ELI-D, QTAIM, density Laplacian and bond-path analyses were visualized using the Paraview program,[33] with the results presented in Fig. S11 and Fig. S12.

Table S2 Metal-metal and metal-ligand internuclear distances (Å) in [**1**]$^{0/+}$ from X-ray data analyses and DFT optimization.^{a}

	**[1]^{0}**		**[1]$^{+}$**	
	X-ray[11]	DFT	X-ray ^{b}[12]	DFT
**Fe–Ni**	2.467	2.459	2.818	2.803
**Fe–S1**	2.284	2.331	2.289	2.328
**Fe–S2**	2.275	2.310	2.296	2.327
**Fe–C1**	1.794	1.772	1.834	1.804
**Fe–C2**	1.800	1.774	1.799	1.728
**Fe–C3**	1.800	1.776	1.790	1.786
**Ni–S1**	2.278	2.349	2.228	2.272
**Ni–S2**	2.231	2.286	2.235	2.261
**Ni–P1**	2.159	2.199	2.187	2.230
**Ni–P2**	2.146	2.197	2.191	2.224

^{a} Atoms labeled as per Fig. S8.
^{b} In absence of X-ray data for [(CO)$_3$Fe(pdt)Ni(dppe)]$^{+}$ ([**1**]$^{+}$), data for a very similar complex [(CO)$_3$Fe(pdt)Ni(dcpe)]$^{+}$ is used (Fig. S8), where dcpe has cyclohexyl rings instead of the phenyl rings in dppe.

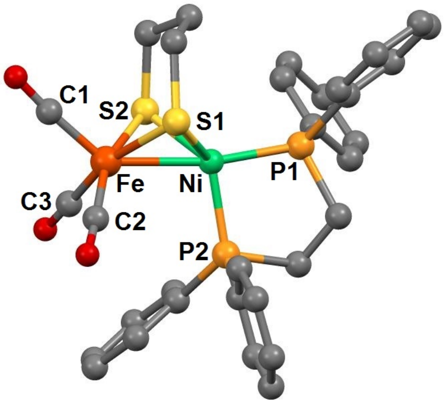

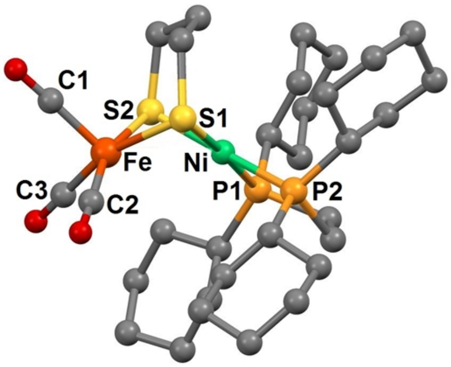

Fig. S8 X-ray structures of **1** (left) and [**1**]$^{+}$ analog [(CO)$_3$Fe(pdt)Ni(dcpe)]$^{+}$ (right).[11,12]

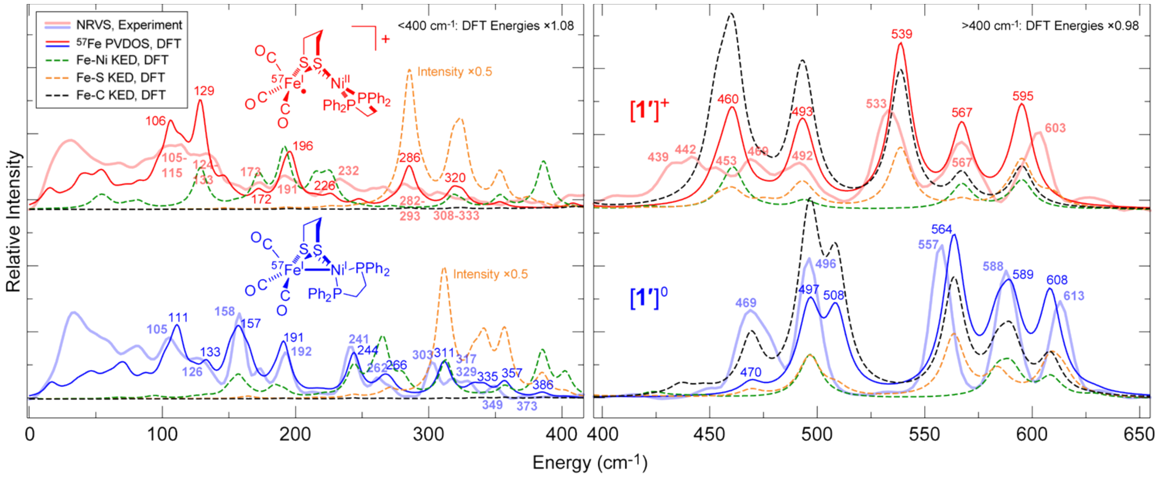

Fig. S9 Observed and DFT calculated NRVS (^{57}Fe PVDOS) data for **1′** (blue, bottom) and [**1′**]$^{+}$ (red, top). The DFT spectra (thin lines) are superimposed with the corresponding experimental spectra in half-transparent (thick lines). Fe-Ni (green), Fe-S (yellow), and Fe-C (black) KED diagrams are provided for both the [**1′**]$^{0/+}$ species, based on the DFT results. The left and right panels correspond to 108% and 98% scaling of the DFT vibrational frequencies in the 0-400 cm^{-1} and 400-650 cm^{-1} regions, respectively. Correspondence between the observed and calculated bands is provided in Table S3.

Table S3 Peak frequencies (cm^{-1}) for experimental NRVS/IR bands of [**1**]$^{0/+}$ and their equivalents from DFT calculations.[a]

	**[1′]^{0}**			**[1′]$^{+}$**		
	**Observed**	*DFT raw*	DFT scaled	**Observed**	*DFT raw*	DFT scaled
**NRVS, 100-400 cm^{-1}** DFT ×1.08	**105**	*103*	111	**105-115** [c]	*98*	106
	**126**	*123*	133	**124-133** [c]	*119*	129
	**158**	*145*	157	**173**	*159*	172
	**192**	*177*	191	**191**	*181*	196
	**241**	*226*	244	**232**	*209*	226
	**262**	*246*	266	**282-293** [c]	*265*	286
	**303**	*288*	311	**308-333** [c]	*296*	320
	**317, 329** [b]	*310*	335			
	**349**	*331*	357			
	**373**	*357*	386			
**NRVS, 400-650 cm^{-1}** DFT ×0.98	**469**	*480*	470	**435-469** [c]	*470*	460
	**496**	*508, 519* [b]	497, 508 [b]	**492**	*504*	493
	**557**	*576*	564	**533**	*551*	539
	**588**	*602*	589	**567**	*579*	567
	**613**	*621*	608	**603**	*608*	595
**IR, 1900-2100 cm^{-1}** DFT ×1.02	**1953**	*1924*	1962	**1988**	*1943*	1981
	**2029**	*1995*	2034	**2057**	*2012*	2051

[a] Corresponding experimental (observed) and calculated (scaled DFT) spectra are provided in Figs. 2, Fig. S9 left (NRVS, 100-400 cm^{-1}), Fig. S9 right (NRVS, 400-650 cm^{-1}), and Fig. S3 (IR, 1900-2100 cm^{-1}).

The band positions rounded to the nearest cm^{-1}. Homogeneous scaling factors were applied to the raw DFT frequencies, as specified in the leftmost column and explained in the text. Animations of the DFT normal modes producing the NRVS/IR bands listed is available as part of the electronic SI.
[b] Correspondence can only be provided between a pair of experimental bands and single calculated band, or vice versa.
[c] Complex set of experimental features matching a single calculated band.

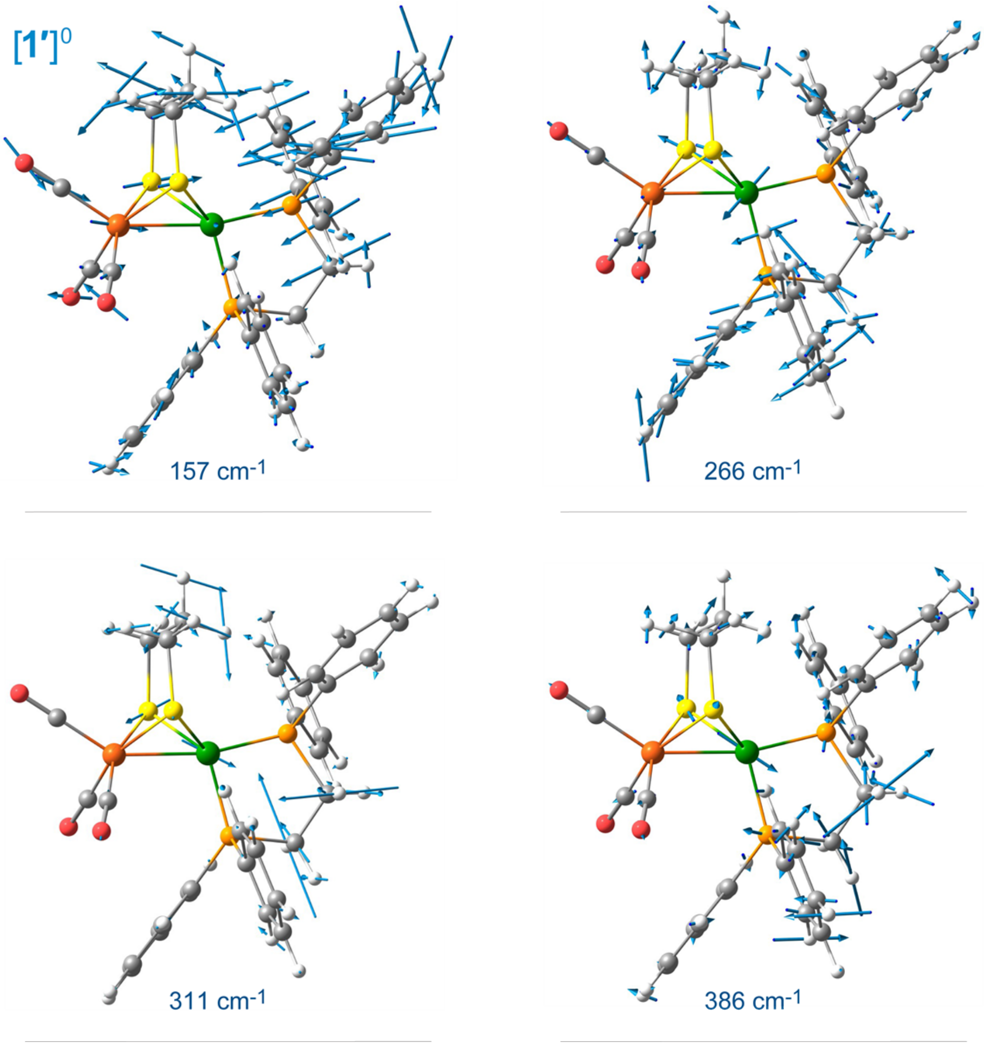

Fig. S10 Scaled arrow depiction of nuclear displacements for selected normal modes with significant Fe-Ni character calculated for **1′** (157, 266, 311, and 386 cm^{-1}). Animated representations of all the significant [**1′**]$^{0/+}$ modes are available in the electronic SI.

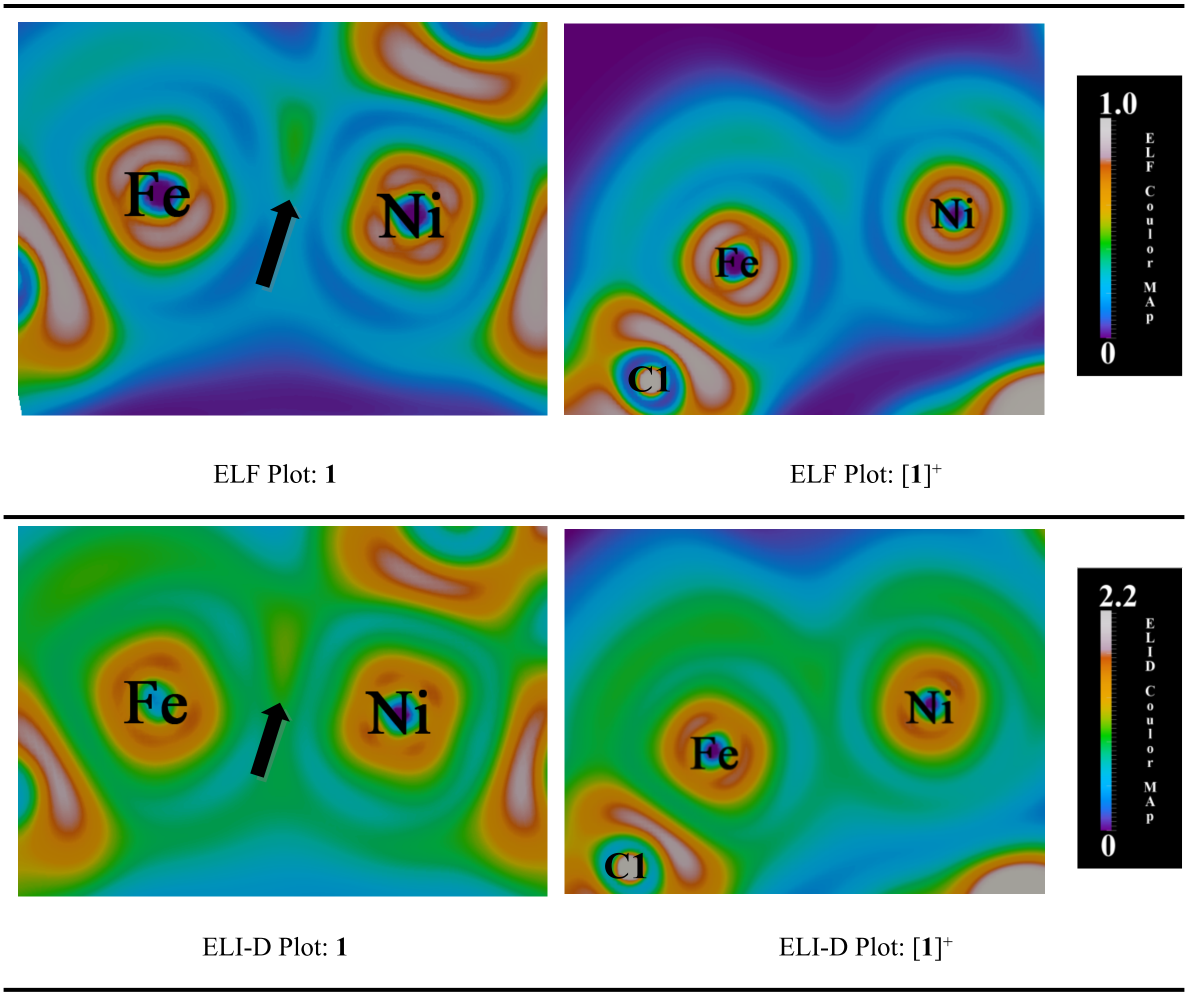

Fig. S11 ELF (top) and ELI-D (bottom) analyses of the Ni–Fe bonding in [**1**]$^{0/+}$ (left/right) with the corresponding color map legends (far right). Ni–Fe bond attractor positions for [**1**]^{0} are indicated by the arrows. Plots are shown in the Fe-Ni-C1 plane with atoms labeled as in Fig. S8.

Red Point = Attractor
Blue Point = Ring Critical Point
Green Point = Saddle Point
Yellow Point = Minimum

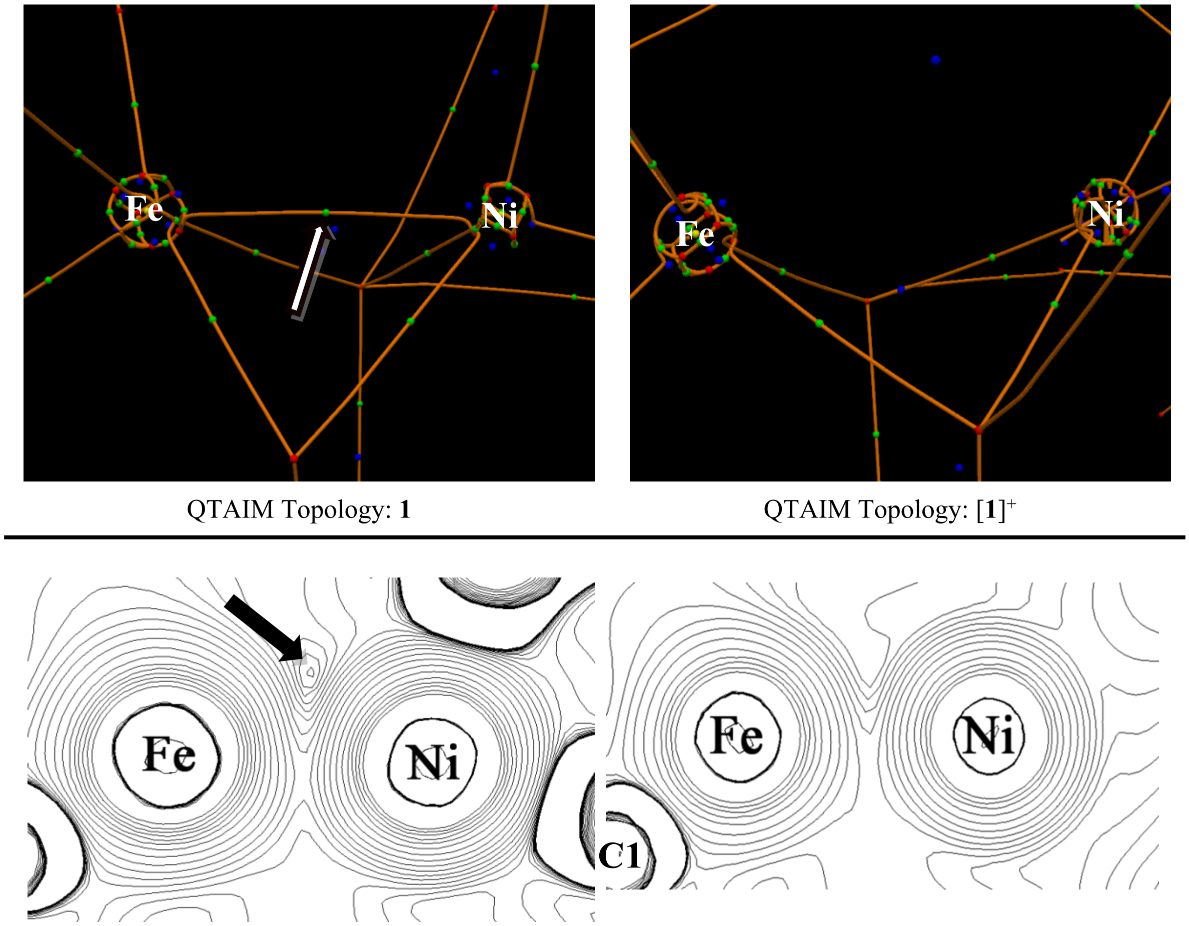

Laplacian Plot: **1**

Laplacian Plot: [**1**]$^{+}$

Fig. S12 QTAIM topologic (top) and total electron density Laplacian (bottom) of Ni–Fe bonding in [**1**]$^{0/+}$ (left/right). The position of the bond critical point and of the Laplacian attractor for [**1**]^{0} are indicated by the arrows. Plots are shown in the Fe-Ni-C1 plane with atoms labeled as in Fig. S8.

References

1 G. G. Nunes, R. C. R. Bottini, D. M. Reis, P. H. C. Camargo, D. J. Evans, P. B. Hitchcock, G. J. Leigh, E. L. Sá, J. F. Soares, *Inorg. Chim. Acta*, 2004, **357**, 1219.

2 M. E. Carroll, J. Chen, D. E. Gray, J. C. Lansing, T. B. Rauchfuss, D. Schilter, P. I. Volkers, S. R. Wilson, *Organometallics*, 2014, **33**, 858.

3 M. Schmidt, G. G. Hoffmann, *J. Organomet. Chem.*, 1977, **124**, c5.

4 D. Schilter, T. B. Rauchfuss, *Dalton Trans.*, 2012, **41**, 13324.

5 B. E.Barton, T. B. Rauchfuss, *J. Am. Chem. Soc.*, 2010, **132**, 14877.

6 Y. Guo, H. Wang, Y. Xiao, S. Vogt, R. K. Thauer, S. Shima, P. I. Volkers, T. B. Rauchfuss, V. Pelmenschikov, D. A. Case, E. Alp, W. Sturhahn, Y. Yoda, S. P. Cramer, *Inorg. Chem.*, 2008, **47**, 3969.

7 S. P. Cramer, Y. Xiao, H. Wang, Y. Guo, M. C. Smith, *Hyperfine Interact.*, 2006, **170**, 47.

8 M. C. Smith, Y. Xiao, H. Wang, S. J. George, D. Coucouvanis, M. Koutmos, W. Sturhahn, E. E. Alp, J. Zhao, S. P. Cramer, *Inorg. Chem.*, 2005, **44**, 5562.

9 W. Sturhahn, T. S. Toellner, E. E. Alp, X. Zhang, M. Ando, Y. Yoda, S. Kikuta, M. Seto, C. W. Kimball, B. Dabrowski, *Phys. Rev. Lett.*, 1995, **74**, 3832.

10 H. Wang, Y. Yoda, S. Kamali, Z.-H. Zhou, S. P. Cramer, *J. Synchrotron Rad.*, 2012, **19**, 257.

11 W. Zhu, W. A. C. Marr, Q. Wang, F. Neese, D. J. E. Spencer, A. J. Blake, P. A. Cooke, C. Wilson, M. Schröder, *Proc. Nat. Acad. Sci. U.S.A.*, 2005, **102**, 18280.

12 D. Schilter, M. J. Nilges, M. Chakrabarti, P. A. Lindahl, T. B. Rauchfuss, M. Stein, *Inorg. Chem.*, 2012, **51**, 2338.

13 M. J. Frisch, G. W. Trucks, H. B. Schlegel, G. E. Scuseria, M. A. Robb, J. R. Cheeseman, G. Scalmani, V. Barone, B. Mennucci, G. A. Petersson, H. Nakatsuji, M. Caricato, X. Li, H. P. Hratchian, A. F. Izmaylov, J. Bloino, G. Zheng, J. L. Sonnenberg, M. Hada, M. Ehara, K. Toyota, R. Fukuda, J. Hasegawa, M. Ishida, T. Nakajima, Y. Honda, O. Kitao, H. Nakai, T. Vreven, J. A. Montgomery, Jr., J. E. Peralta, F. Ogliaro, M. Bearpark, J. J. Heyd, E. Brothers, K. N. Kudin, V. N. Staroverov, R. Kobayashi, J. Normand, K. Raghavachari, A. Rendell, J. C. Burant, S. S. Iyengar, J. Tomasi, M. Cossi, N. Rega, J. M. Millam, M. Klene, J. E. Knox, J. B. Cross, V. Bakken, C. Adamo, J. Jaramillo, R. Gomperts, R. E. Stratmann, O. Yazyev, A. J. Austin, R. Cammi, C. Pomelli, J. W. Ochterski, R. L. Martin, K. Morokuma, V. G. Zakrzewski, G. A. Voth, P. Salvador, J. J. Dannenberg, S. Dapprich, A. D. Daniels, Ö. Farkas, J. B. Foresman, J. V. Ortiz, J. Cioslowski, D. J. Fox, *Gaussian 09*, Revision A.02, Gaussian Inc., Wallingford CT, 2009.

14 *Jaguar*, version 7.9, Schrödinger, LLC, New York, NY, 2012

15 A. D. Becke, *Phys. Rev. A*, 1988, **38**, 3098.

16 J. P. Perdew, *Phys. Rev. B*, 1986, **33**, 8822.

17 J. Tomasi, B. Mennucci, R. Cammi, *Chem. Rev.*, 2005, **105**, 2999.

18 D. Mitra, S. J. George, Y. S. Guo, S. Kamali, S. Keable, J. W. Peters, V. Pelmenschikov, D. A. Case, S. P. Cramer, *J. Am. Chem. Soc.* 2013, **135**, 2530.

19 L. F. Yan, V. Pelmenschikov, C. H. Dapper, A. D. Scott, W. E. Newton, S. P. Cramer, *Chem. Eur. J.*, 2012, **18**, 16349.

20 V. Pelmenschikov, Y. S. Guo, H. Wang, S. P. Cramer, D. A. Case, *Faraday*

Discuss., 2011, **148**, 409.
21 D. Mitra, V. Pelmenschikov, Y. S. Guo, D. A. Case, H. Wang, W. B. Dong, M. L. Tan, T. Ichiye, F. E. Jenney, M. W. W. Adams, Y. Yoda, J. Y. Zhao, S. P. Cramer, *Biochemistry*, 2011, **50**, 5220.
22 H. S. Shafaat, K. Weber, T. Petrenko, F. Neese, W. Lubitz, *Inorg. Chem.*, 2012, **51**, 11787.
23 J. P. Perdew, K. Burke, M. Ernzerhof, *Phys. Rev. Lett.*, 1996, **77**, 3865.
24 J. P. Perdew, K. Burke, M. Ernzerhof, *Phys. Rev. Lett.*, 1997, **78**, 1396.
25 C. T. Lee, W. T. Yang, R. G. Parr, *Phys. Rev. B*, 1988, **37**, 785.
26 A. D. Becke, *J. Chem. Phys.*, 1993, **98**, 5648.
27 S. Grimme, J. Antony, S. Ehrlich, H. Krieg, *J. Chem. Phys.*, 2010, **132**, 154104.
28 L. Goerigk, S. Grimme, *Phys. Chem. Chem. Phys.*, 2011, **13**, 6670.
29 M. Kohout, DGrid, version 4.6, Radebeul, **2011**.
30 A. D. Becke, K. E. Edgecombe, *J. Chem. Phys.* **1990**, *92*, 5397; A. Savin, O. Jepsen, J. Flad, O. K. Andersen, H. Preuss, H. G. Vonschnering, *Angew. Chem. Int. Ed.* **1992**, *31*, 187; M. Kohout, A. Savin, *Int. J. Quantum. Chem.* **1996**, *60*, 875.
31 M. Kohout, *Int. J. Quantum. Chem.* **2004**, *97*, 651; M. Kohout, K. Pernal, F. R. Wagner, Y. Grin, *Theor. Chem. Acc.* **2004**, *112*, 453.
32 R. F. W. Bader, *Atoms in Molecules: A Quantum Theory*, Oxford University Press, Oxford, **1990**.
33 Paraview, version 4.1, Kitware Inc., Clifton Park, New York, USA, **2014**; available from http://www.paraview.org.
