# Supplementary figures and images for: Synthesis and vibrational spectroscopy of 57Fe-labeled models of [NiFe] hydrogenase: first direct observation of a nickel–iron interaction
Source: Chem Commun (Camb). 2014 Sep 19;50(88):13469–72. doi: 10.1039/c4cc04572f (PMC4191989; doi:10.1039/c4cc04572f)

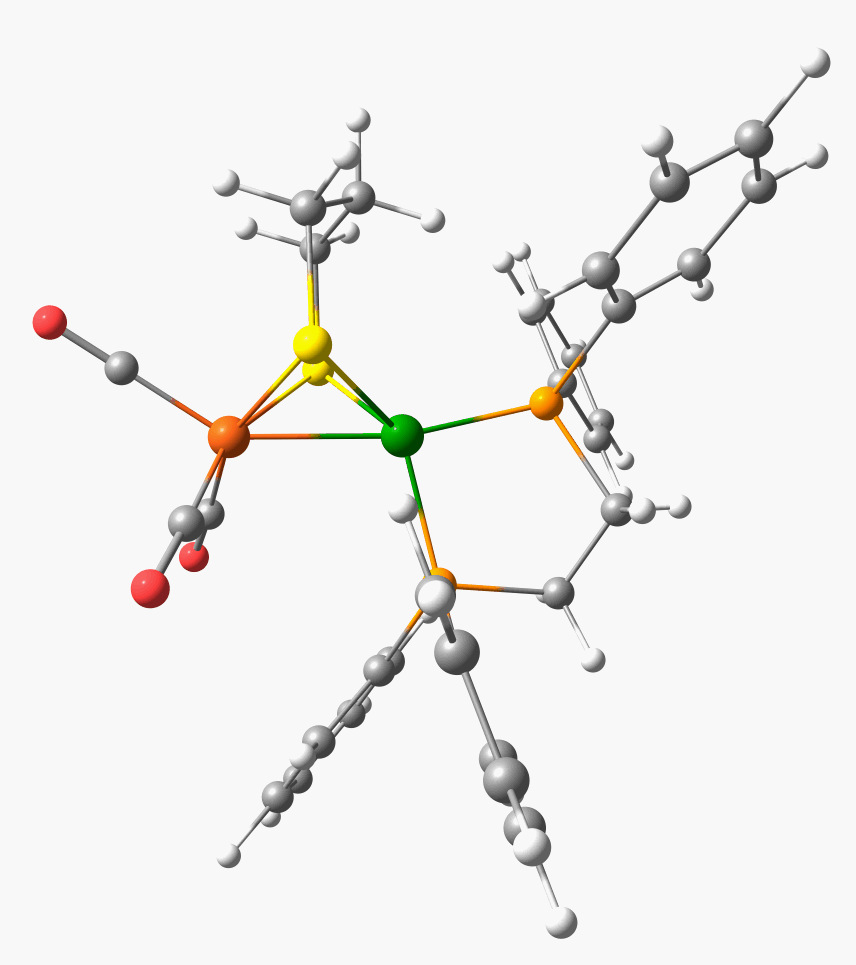

Supplement: Supplementary file 2 [file CC-050-C4CC04572F-s002.zip › DFT-calculated modes for 1' and [1']+/DFT-calculated modes for 1'/1prime-zero_DFT_111cm-1.gif]

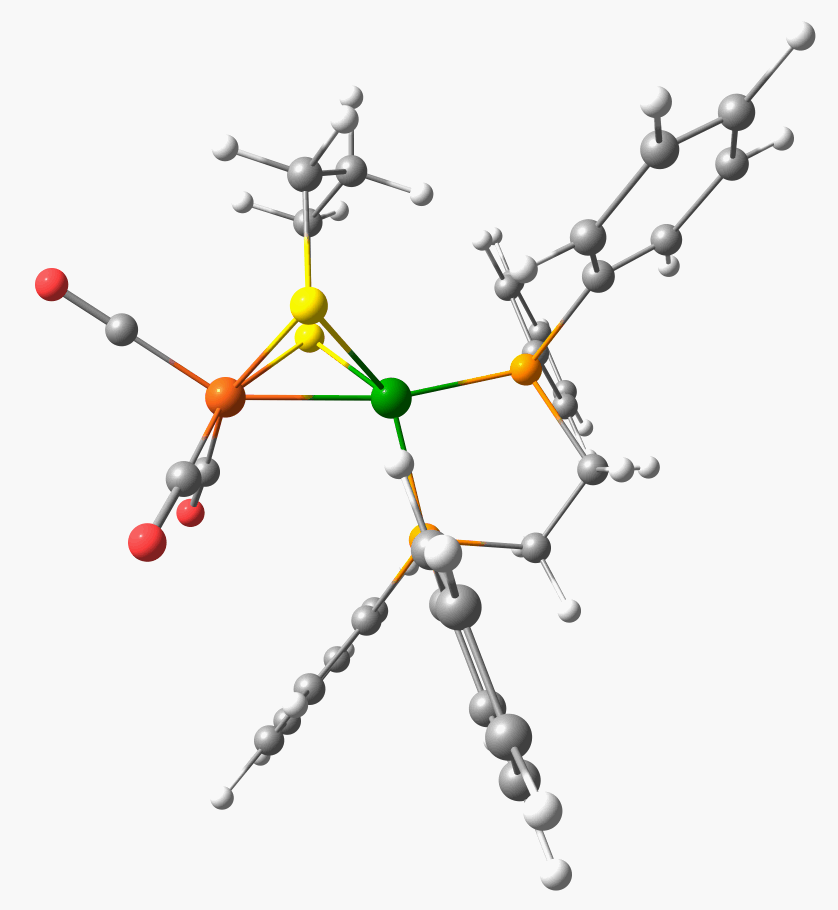

Supplement: Supplementary file 2 [file CC-050-C4CC04572F-s002.zip › DFT-calculated modes for 1' and [1']+/DFT-calculated modes for 1'/1prime-zero_DFT_133cm-1.gif]

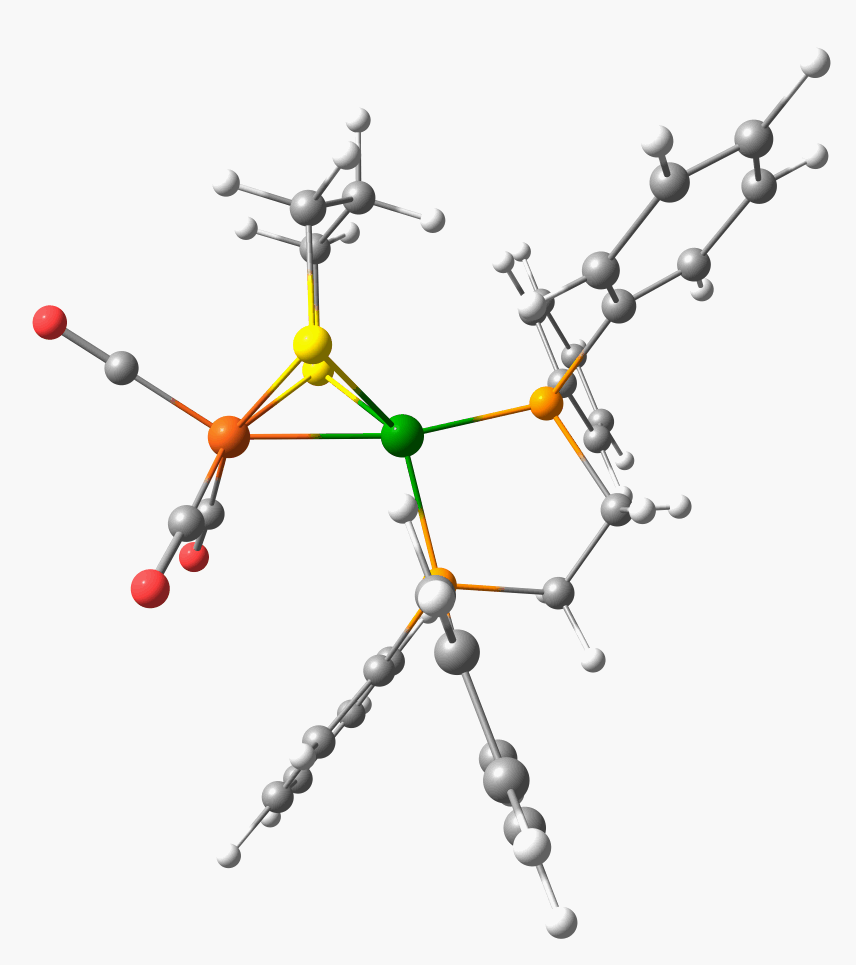

Supplement: Supplementary file 2 [file CC-050-C4CC04572F-s002.zip › DFT-calculated modes for 1' and [1']+/DFT-calculated modes for 1'/1prime-zero_DFT_157cm-1.gif]

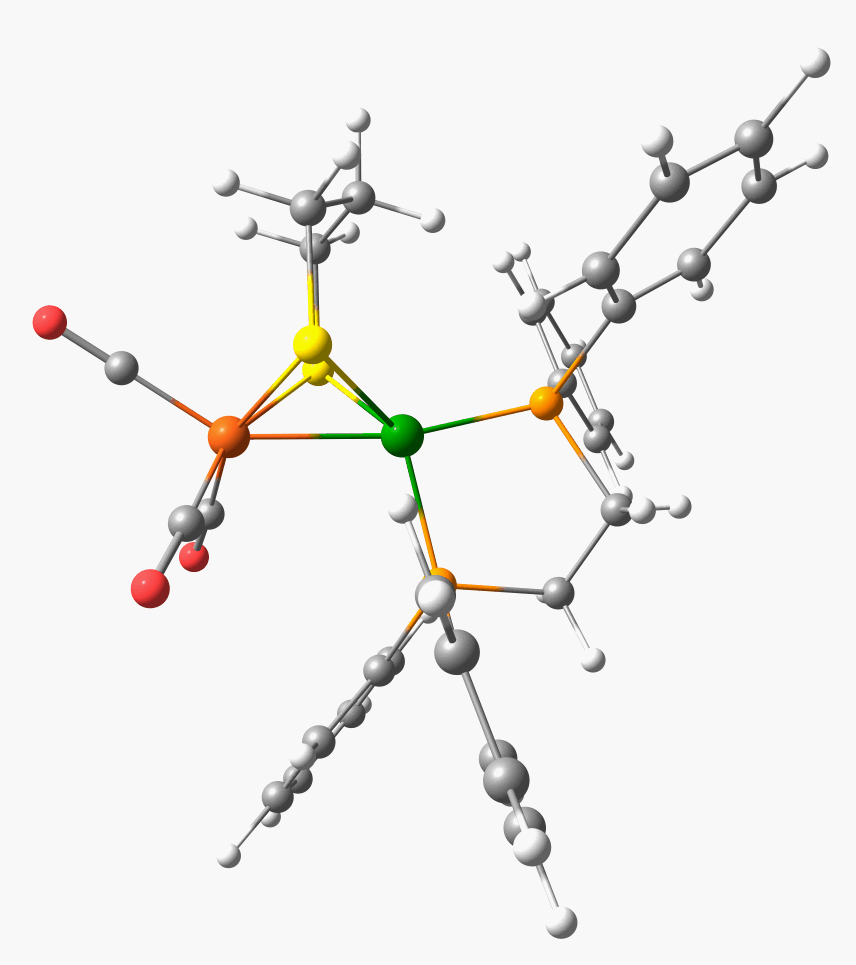

Supplement: Supplementary file 2 [file CC-050-C4CC04572F-s002.zip › DFT-calculated modes for 1' and [1']+/DFT-calculated modes for 1'/1prime-zero_DFT_191cm-1.gif]

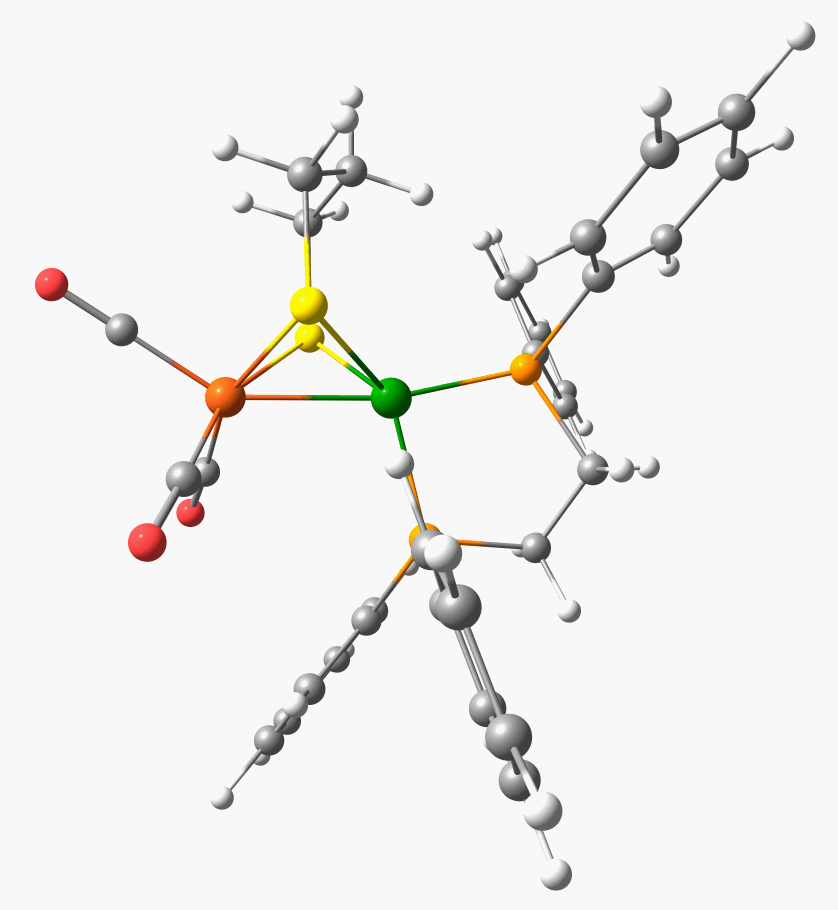

Supplement: Supplementary file 2 [file CC-050-C4CC04572F-s002.zip › DFT-calculated modes for 1' and [1']+/DFT-calculated modes for 1'/1prime-zero_DFT_1962cm-1.gif]

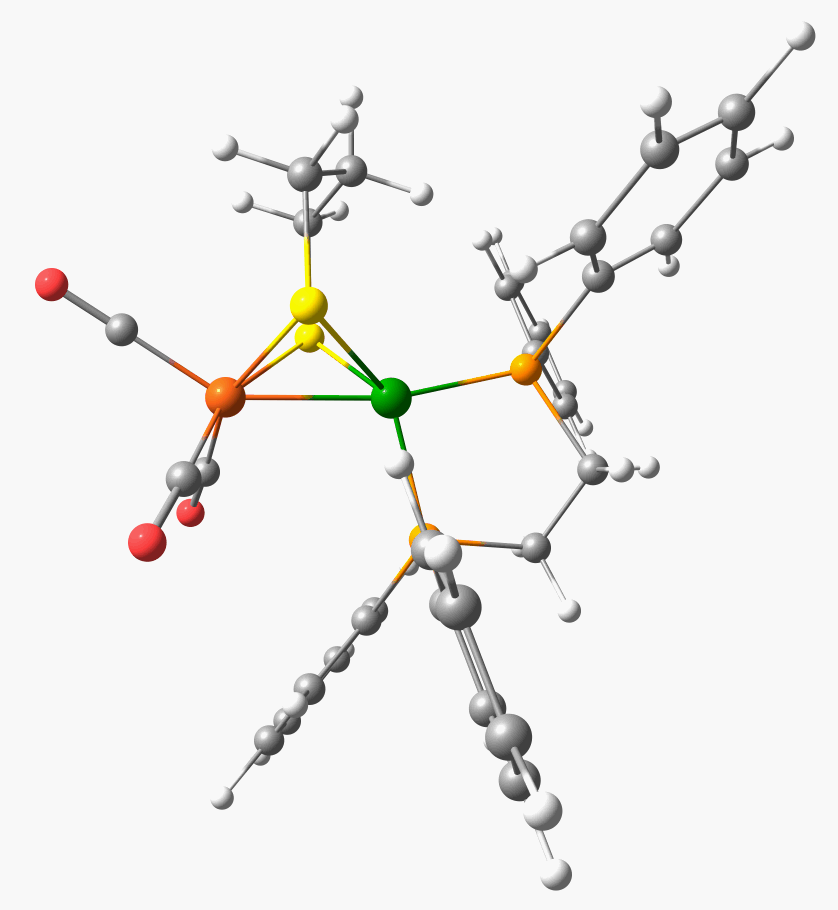

Supplement: Supplementary file 2 [file CC-050-C4CC04572F-s002.zip › DFT-calculated modes for 1' and [1']+/DFT-calculated modes for 1'/1prime-zero_DFT_2034cm-1.gif]

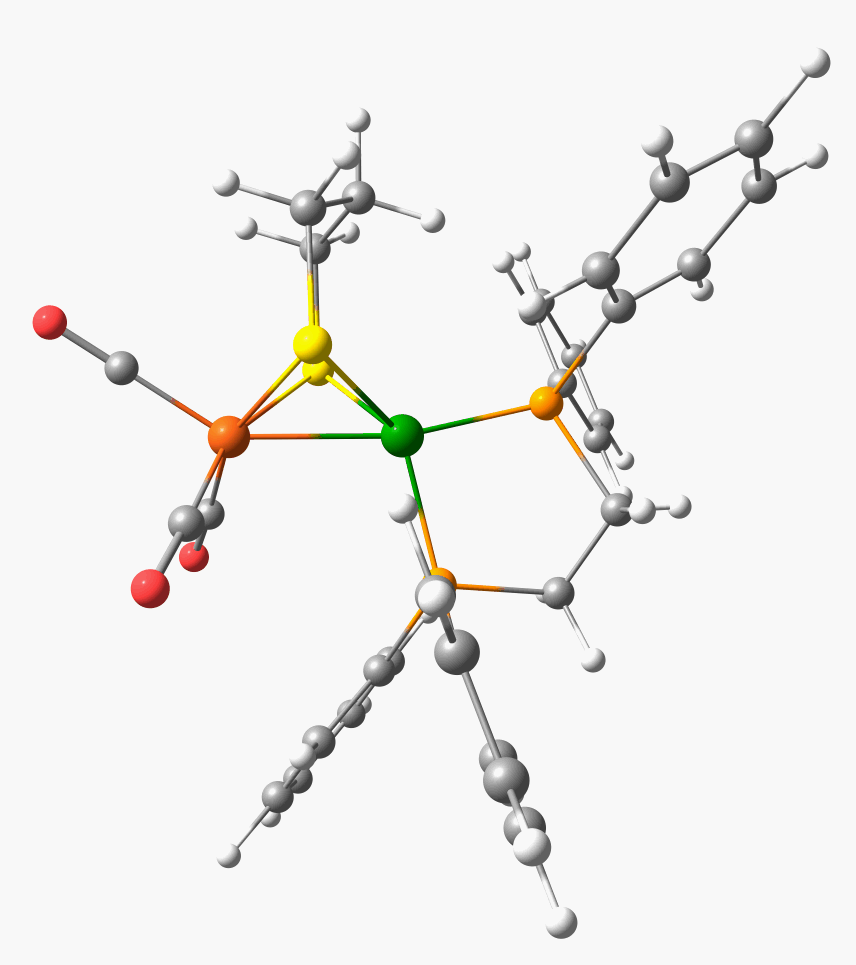

Supplement: Supplementary file 2 [file CC-050-C4CC04572F-s002.zip › DFT-calculated modes for 1' and [1']+/DFT-calculated modes for 1'/1prime-zero_DFT_244cm-1.gif]

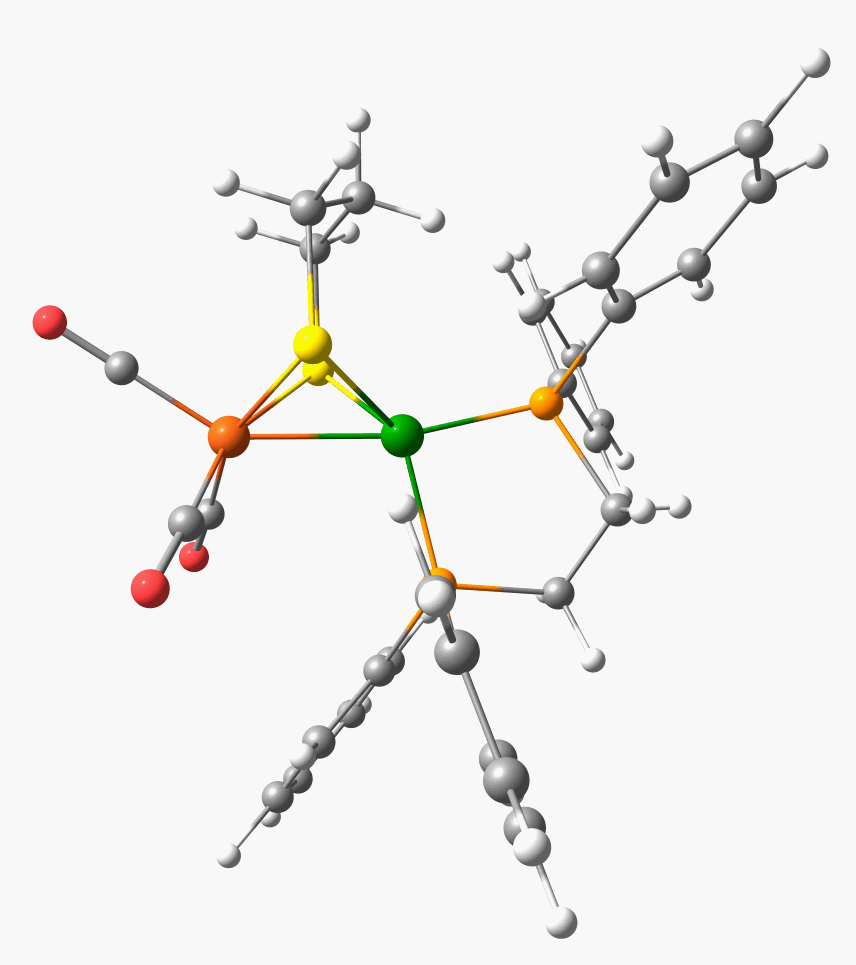

Supplement: Supplementary file 2 [file CC-050-C4CC04572F-s002.zip › DFT-calculated modes for 1' and [1']+/DFT-calculated modes for 1'/1prime-zero_DFT_266cm-1.gif]

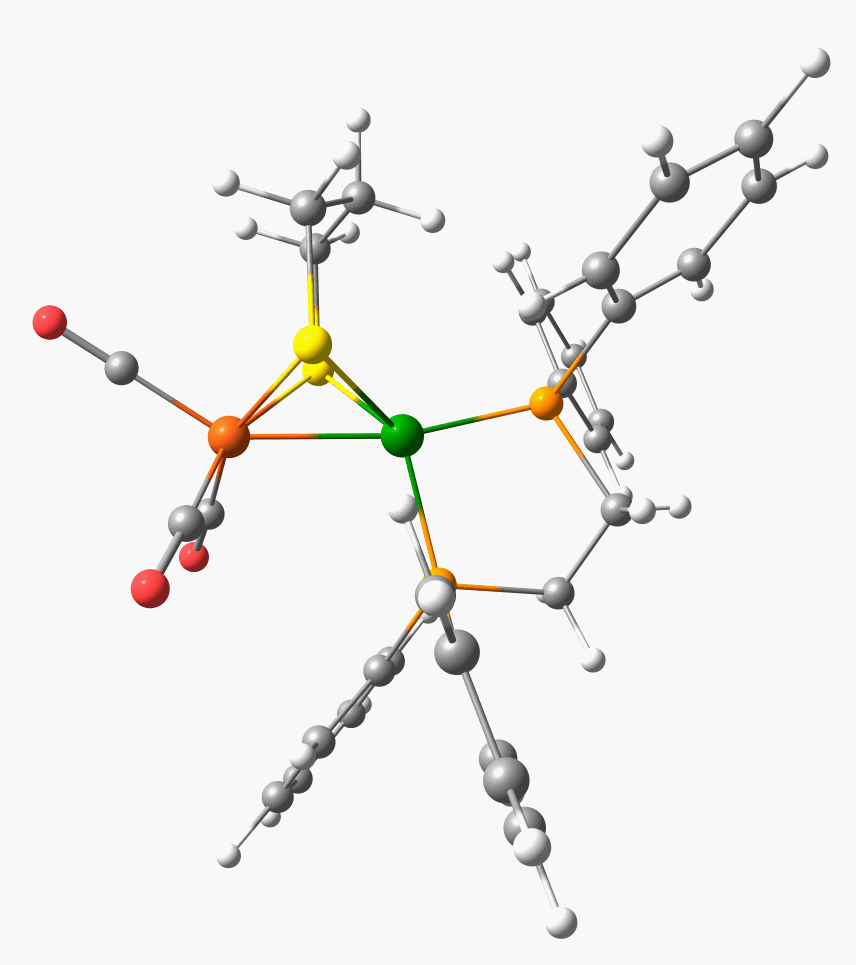

Supplement: Supplementary file 2 [file CC-050-C4CC04572F-s002.zip › DFT-calculated modes for 1' and [1']+/DFT-calculated modes for 1'/1prime-zero_DFT_311cm-1.gif]

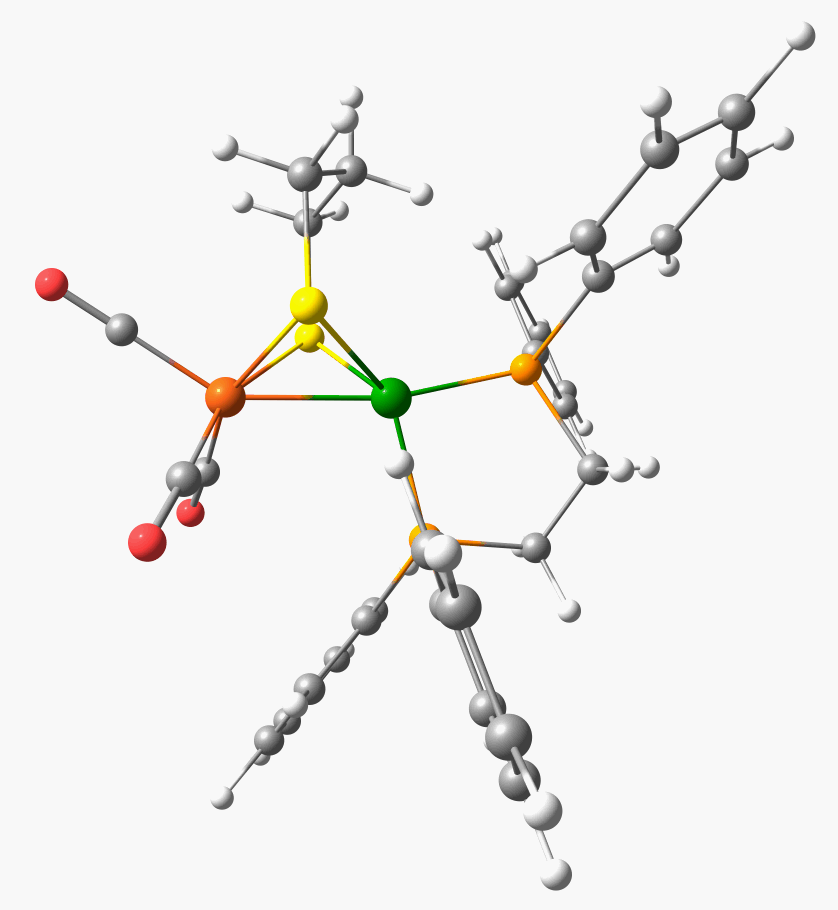

Supplement: Supplementary file 2 [file CC-050-C4CC04572F-s002.zip › DFT-calculated modes for 1' and [1']+/DFT-calculated modes for 1'/1prime-zero_DFT_335cm-1.gif]

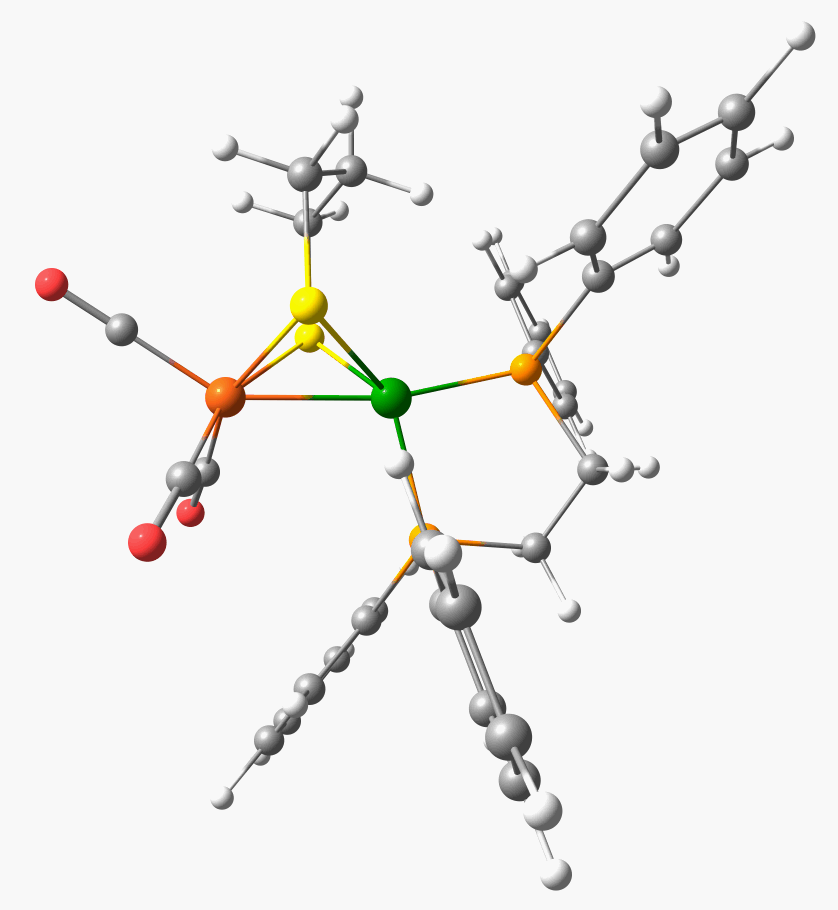

Supplement: Supplementary file 2 [file CC-050-C4CC04572F-s002.zip › DFT-calculated modes for 1' and [1']+/DFT-calculated modes for 1'/1prime-zero_DFT_357cm-1.gif]

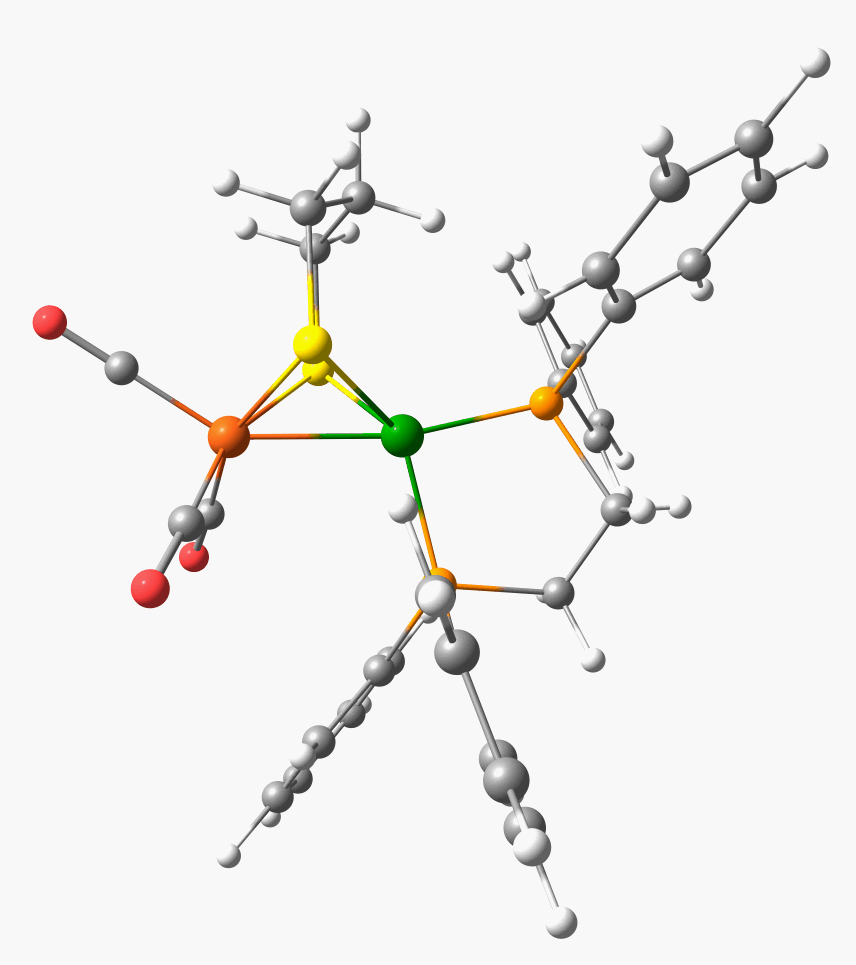

Supplement: Supplementary file 2 [file CC-050-C4CC04572F-s002.zip › DFT-calculated modes for 1' and [1']+/DFT-calculated modes for 1'/1prime-zero_DFT_386cm-1.gif]

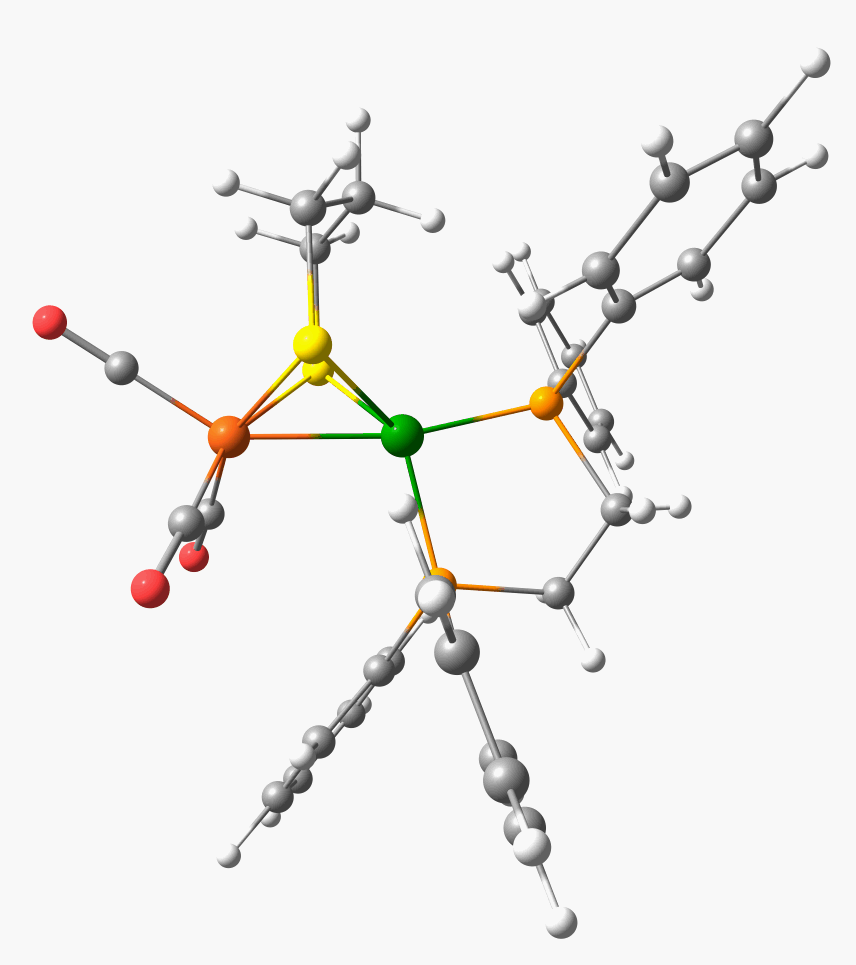

Supplement: Supplementary file 2 [file CC-050-C4CC04572F-s002.zip › DFT-calculated modes for 1' and [1']+/DFT-calculated modes for 1'/1prime-zero_DFT_470cm-1.gif]

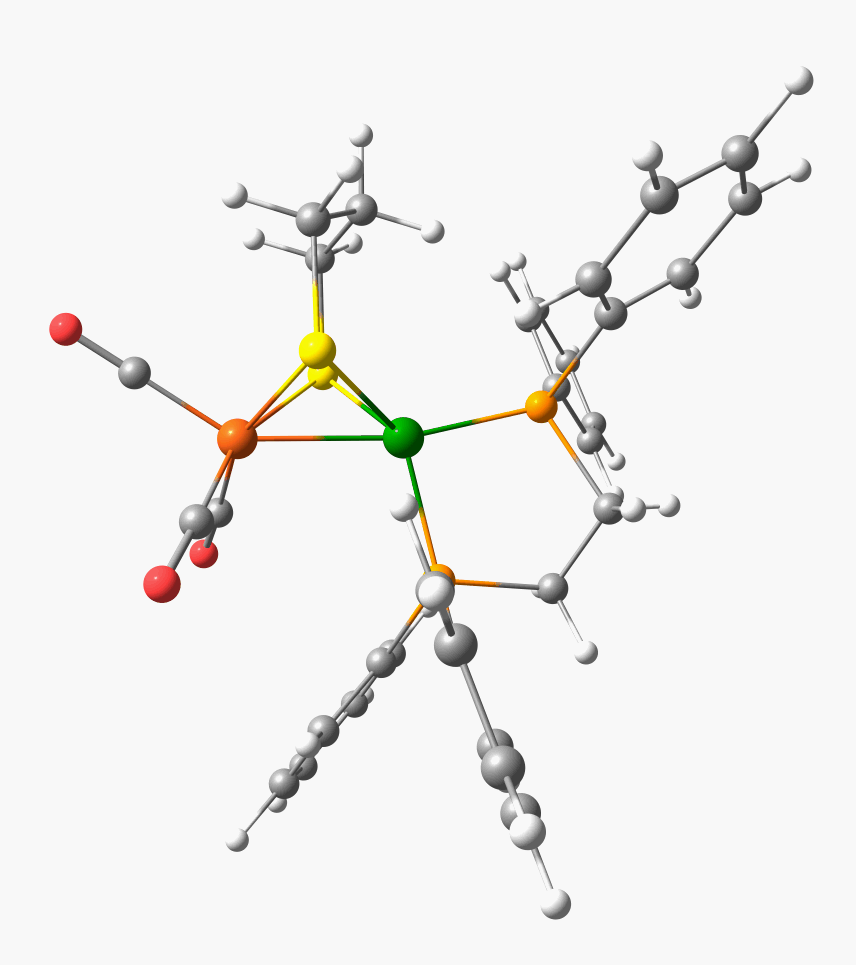

Supplement: Supplementary file 2 [file CC-050-C4CC04572F-s002.zip › DFT-calculated modes for 1' and [1']+/DFT-calculated modes for 1'/1prime-zero_DFT_497cm-1.gif]

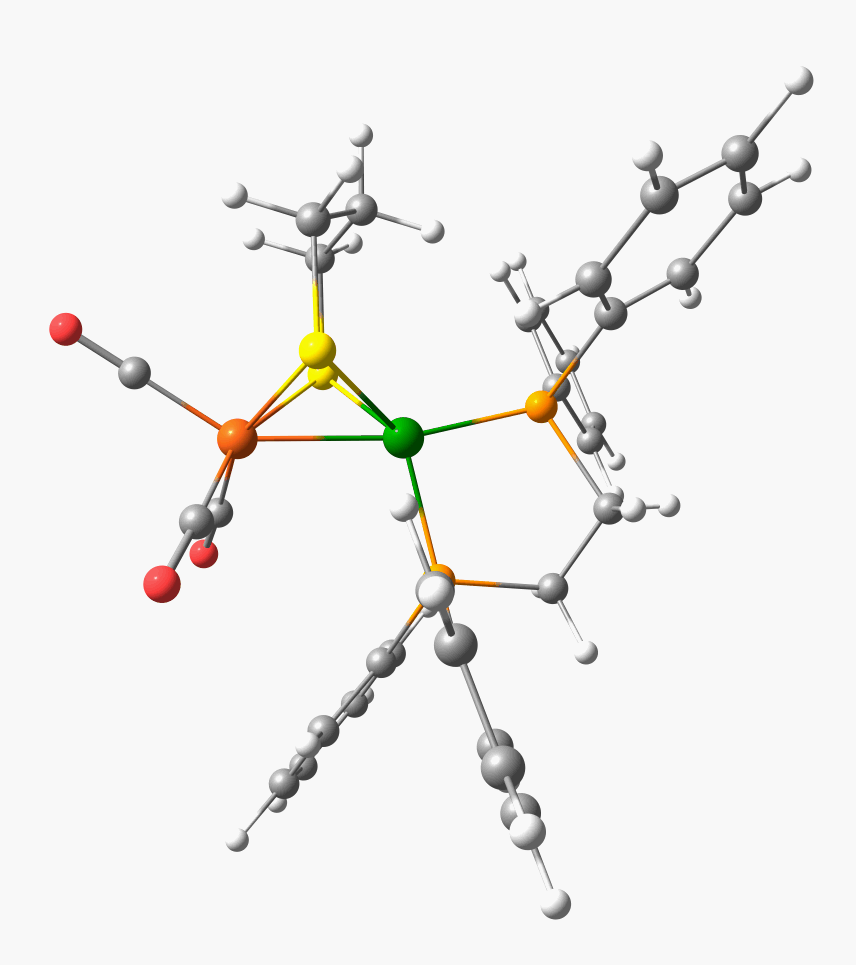

Supplement: Supplementary file 2 [file CC-050-C4CC04572F-s002.zip › DFT-calculated modes for 1' and [1']+/DFT-calculated modes for 1'/1prime-zero_DFT_508cm-1.gif]

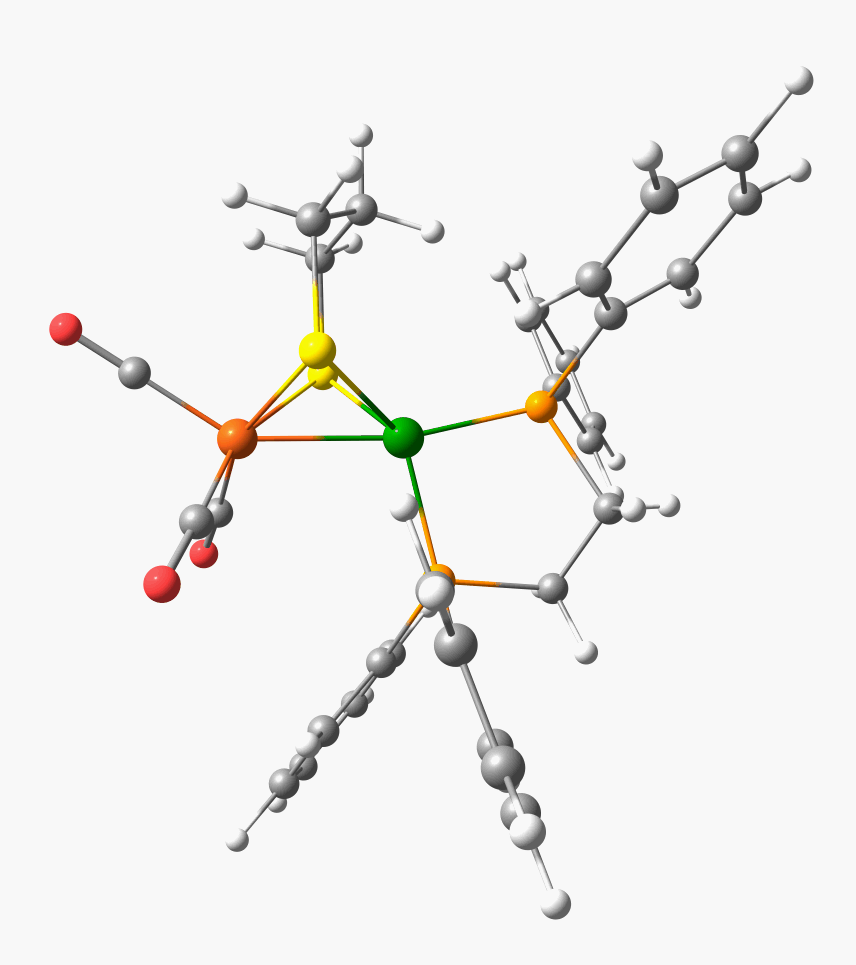

Supplement: Supplementary file 2 [file CC-050-C4CC04572F-s002.zip › DFT-calculated modes for 1' and [1']+/DFT-calculated modes for 1'/1prime-zero_DFT_564cm-1.gif]

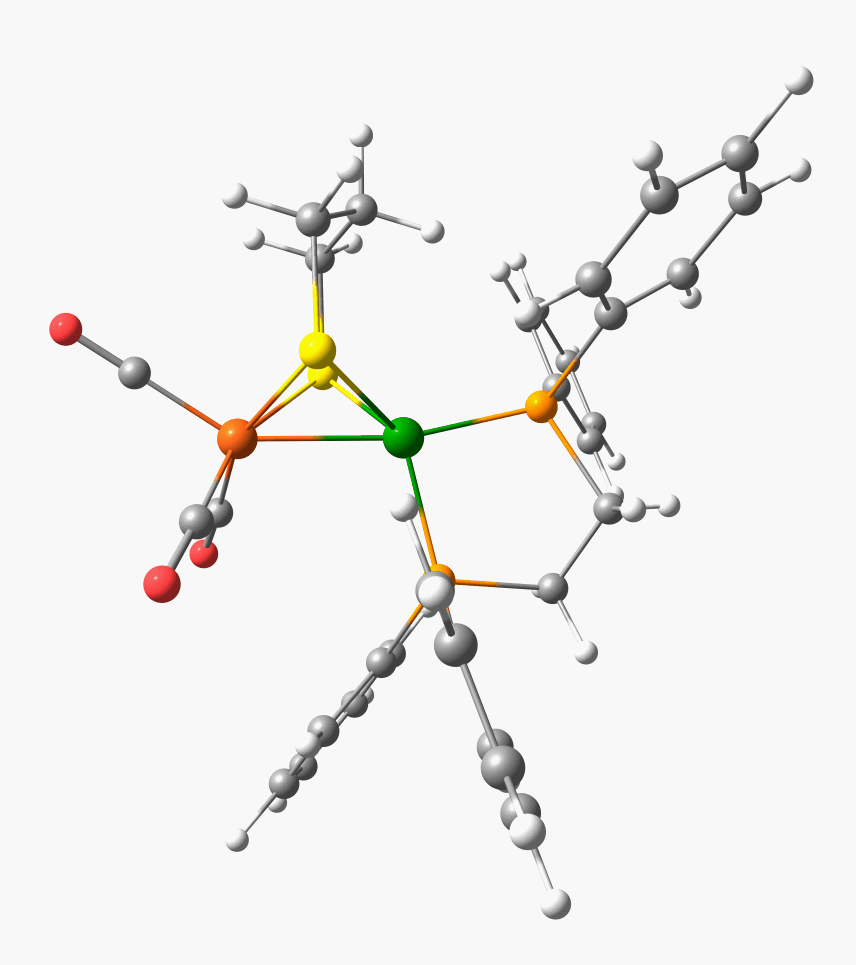

Supplement: Supplementary file 2 [file CC-050-C4CC04572F-s002.zip › DFT-calculated modes for 1' and [1']+/DFT-calculated modes for 1'/1prime-zero_DFT_589cm-1.gif]

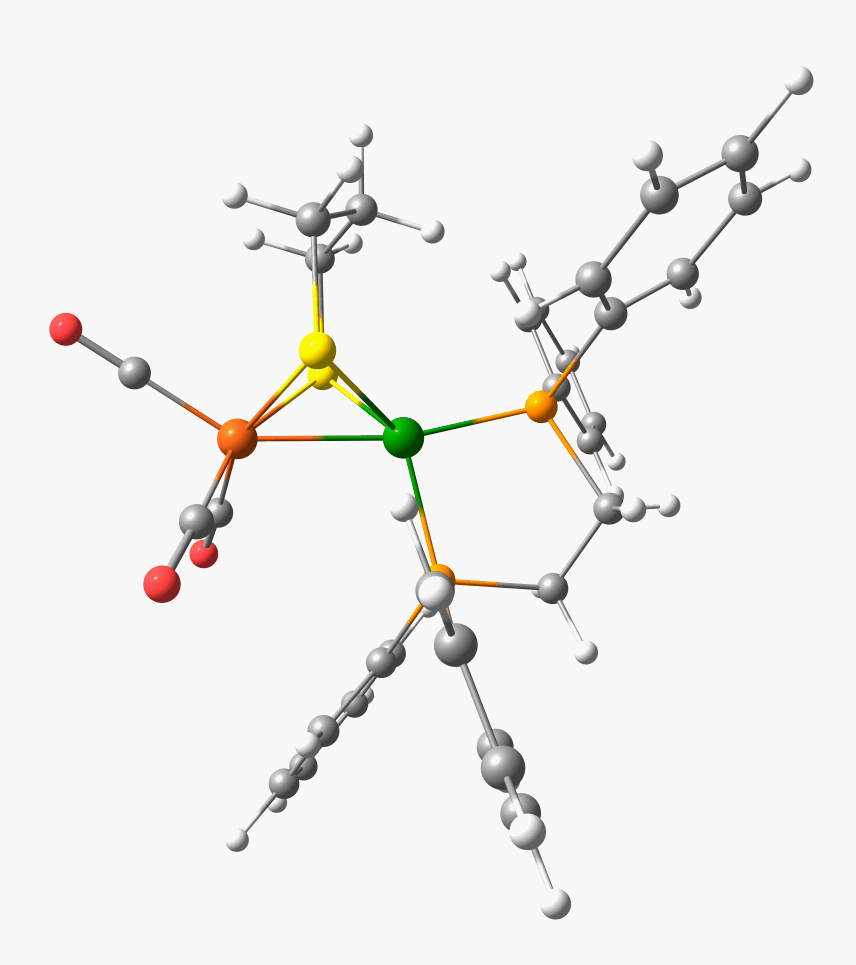

Supplement: Supplementary file 2 [file CC-050-C4CC04572F-s002.zip › DFT-calculated modes for 1' and [1']+/DFT-calculated modes for 1'/1prime-zero_DFT_608cm-1.gif]

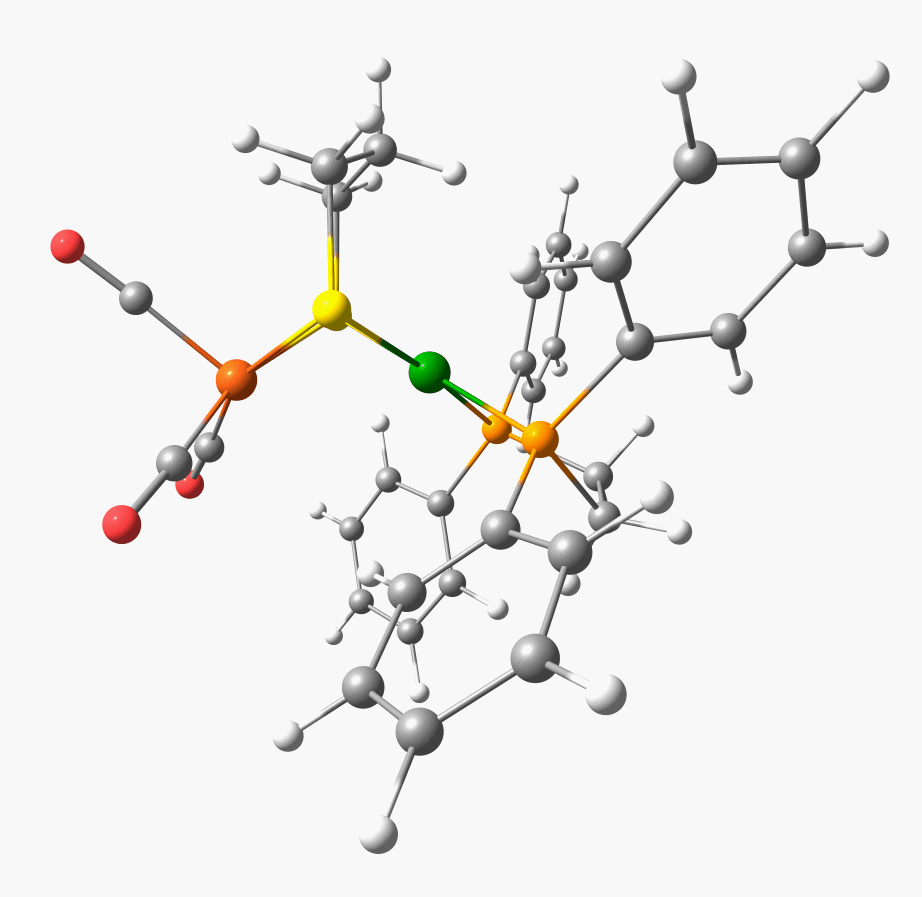

Supplement: Supplementary file 2 [file CC-050-C4CC04572F-s002.zip › DFT-calculated modes for 1' and [1']+/DFT-calculated modes for [1']+/1prime-plus_DFT_106cm-1.gif]

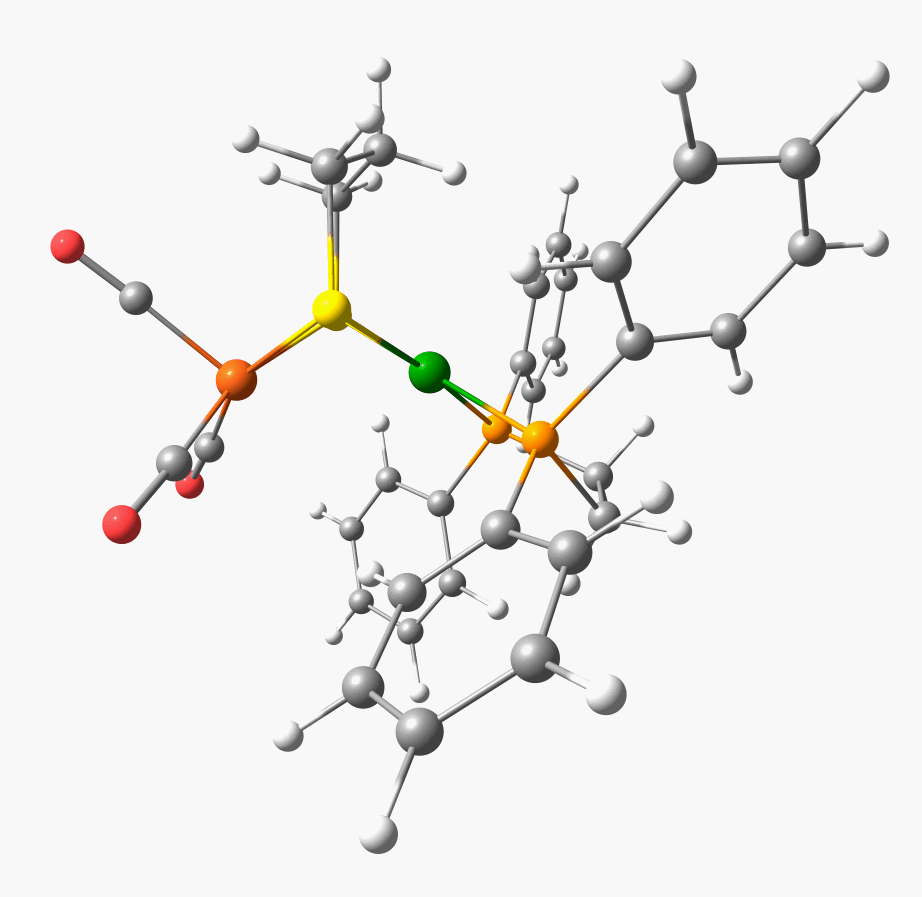

Supplement: Supplementary file 2 [file CC-050-C4CC04572F-s002.zip › DFT-calculated modes for 1' and [1']+/DFT-calculated modes for [1']+/1prime-plus_DFT_129cm-1.gif]

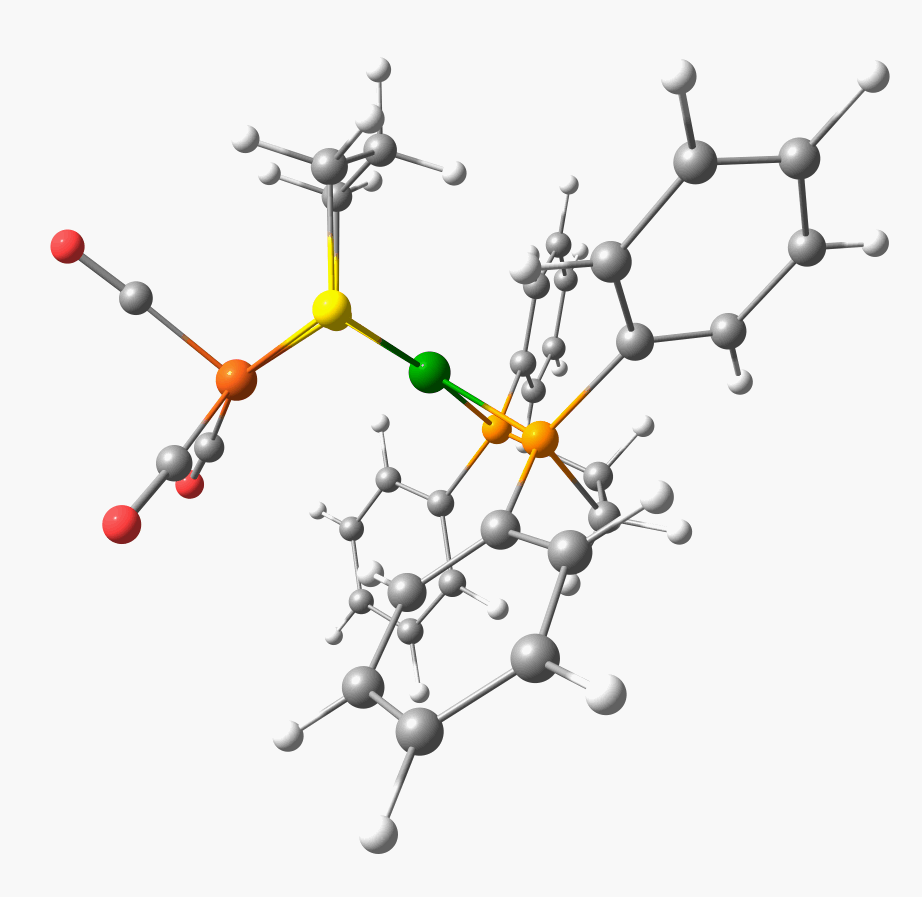

Supplement: Supplementary file 2 [file CC-050-C4CC04572F-s002.zip › DFT-calculated modes for 1' and [1']+/DFT-calculated modes for [1']+/1prime-plus_DFT_172cm-1.gif]

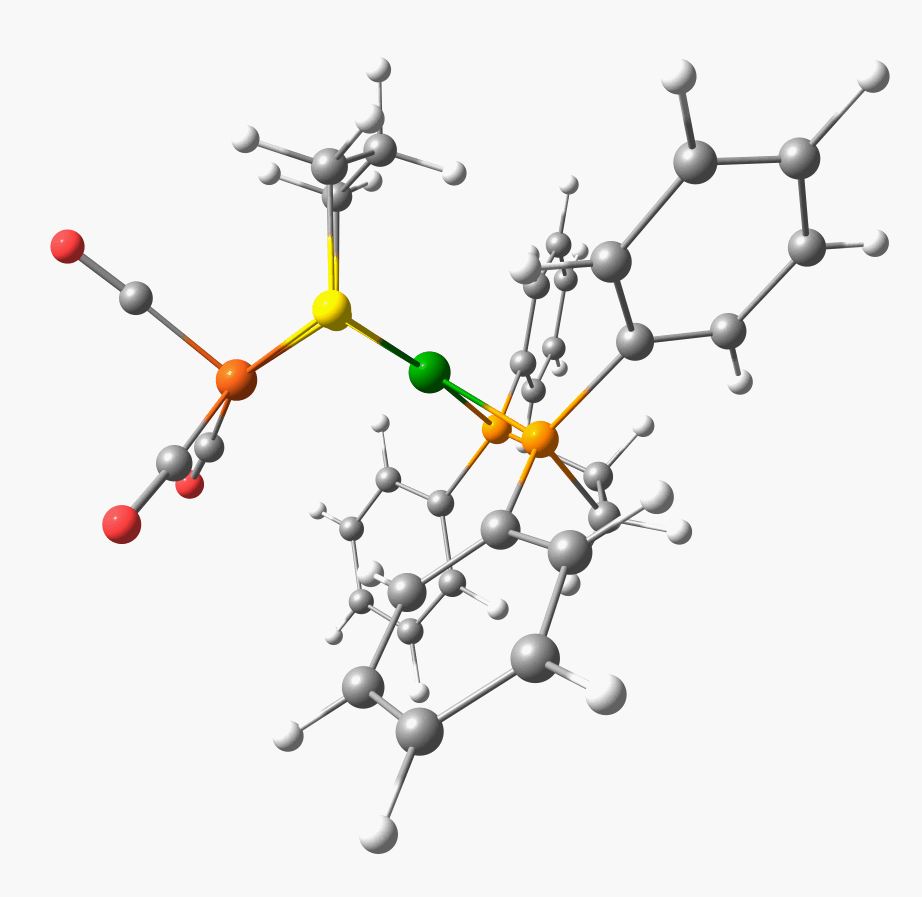

Supplement: Supplementary file 2 [file CC-050-C4CC04572F-s002.zip › DFT-calculated modes for 1' and [1']+/DFT-calculated modes for [1']+/1prime-plus_DFT_196cm-1.gif]

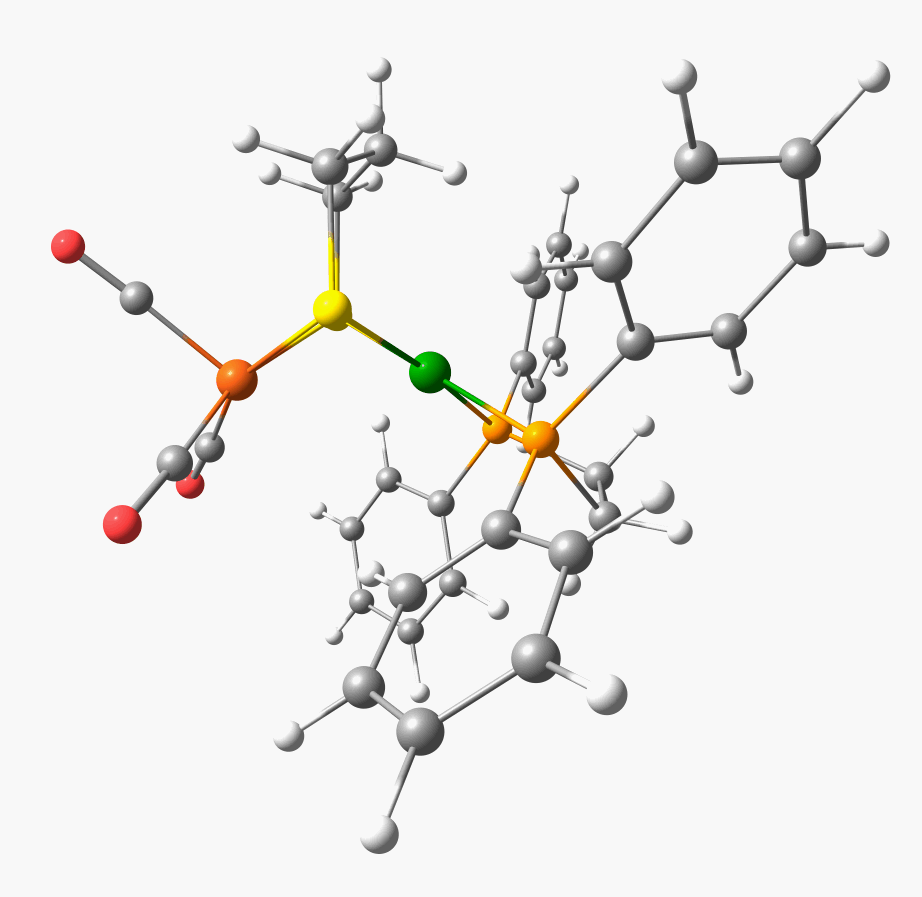

Supplement: Supplementary file 2 [file CC-050-C4CC04572F-s002.zip › DFT-calculated modes for 1' and [1']+/DFT-calculated modes for [1']+/1prime-plus_DFT_1981cm-1.gif]

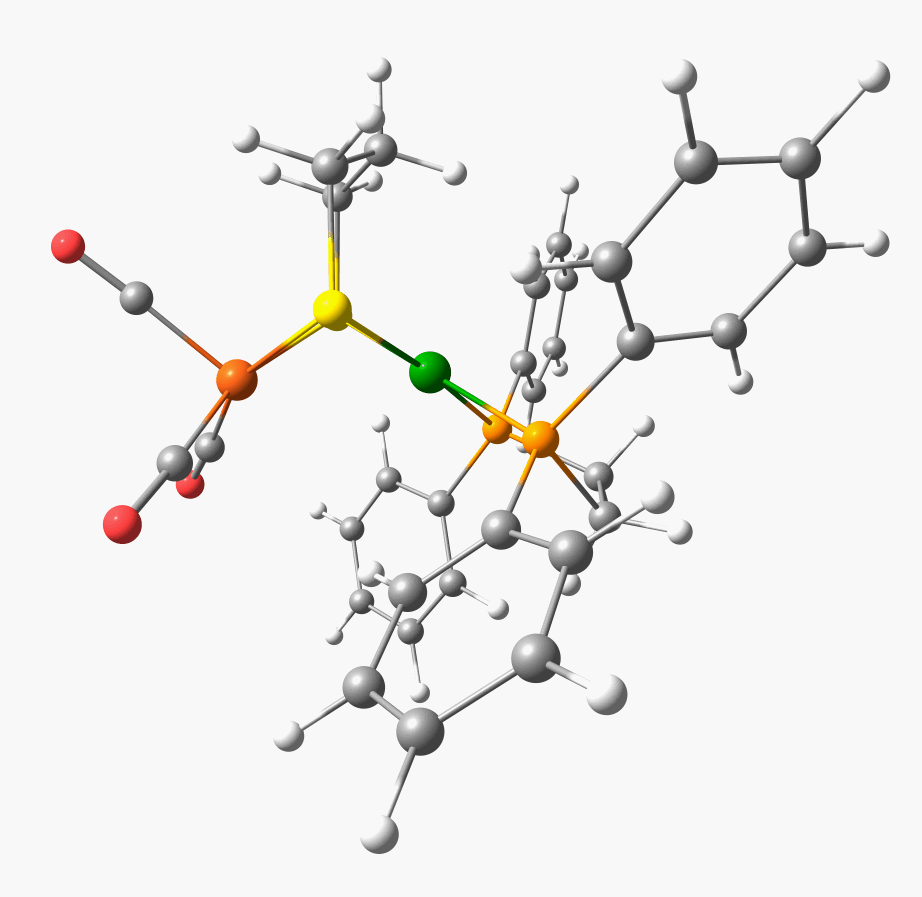

Supplement: Supplementary file 2 [file CC-050-C4CC04572F-s002.zip › DFT-calculated modes for 1' and [1']+/DFT-calculated modes for [1']+/1prime-plus_DFT_2051cm-1.gif]

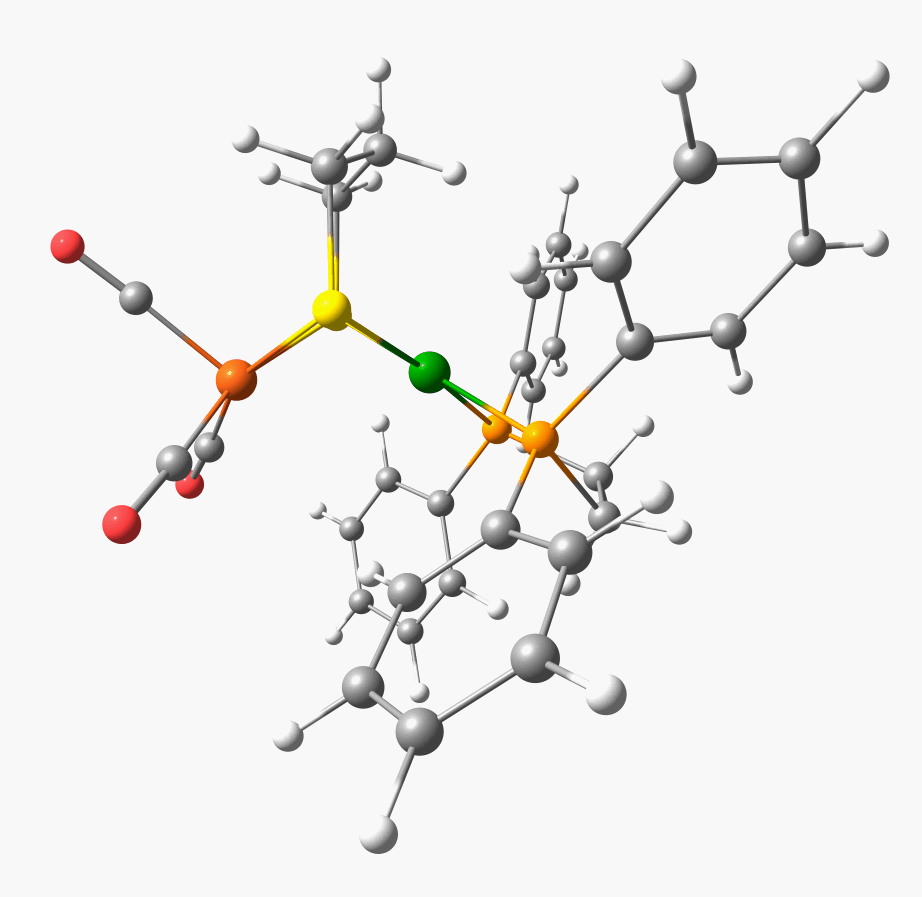

Supplement: Supplementary file 2 [file CC-050-C4CC04572F-s002.zip › DFT-calculated modes for 1' and [1']+/DFT-calculated modes for [1']+/1prime-plus_DFT_226cm-1.gif]

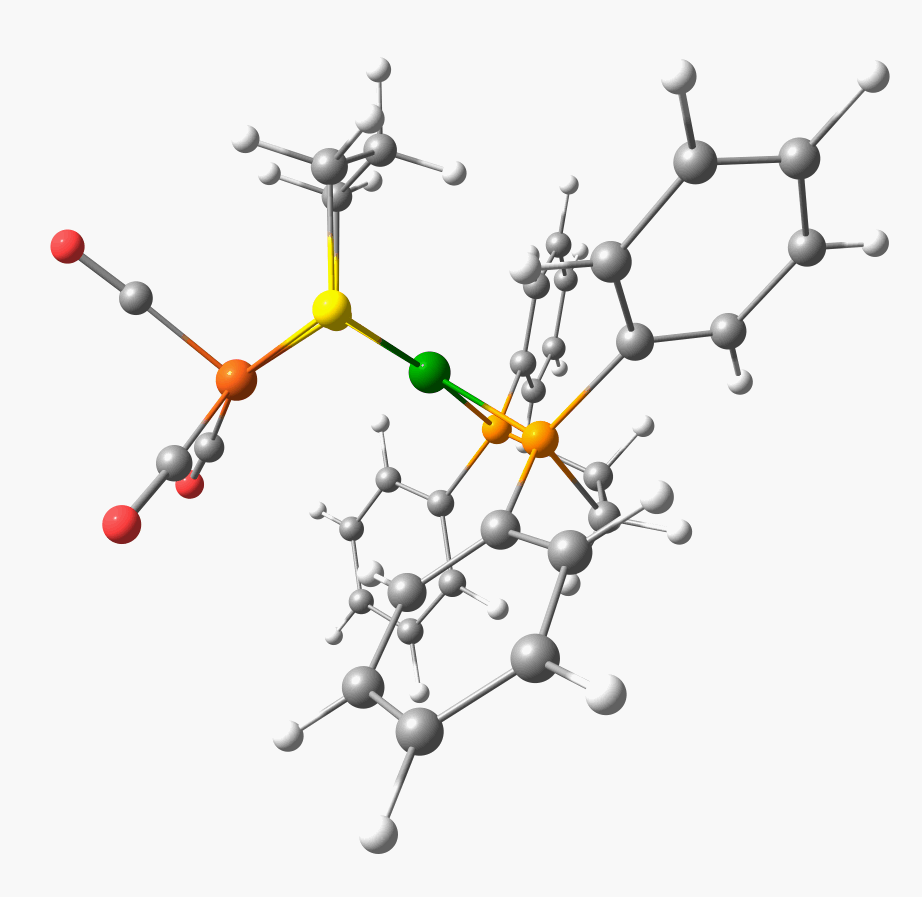

Supplement: Supplementary file 2 [file CC-050-C4CC04572F-s002.zip › DFT-calculated modes for 1' and [1']+/DFT-calculated modes for [1']+/1prime-plus_DFT_286cm-1.gif]

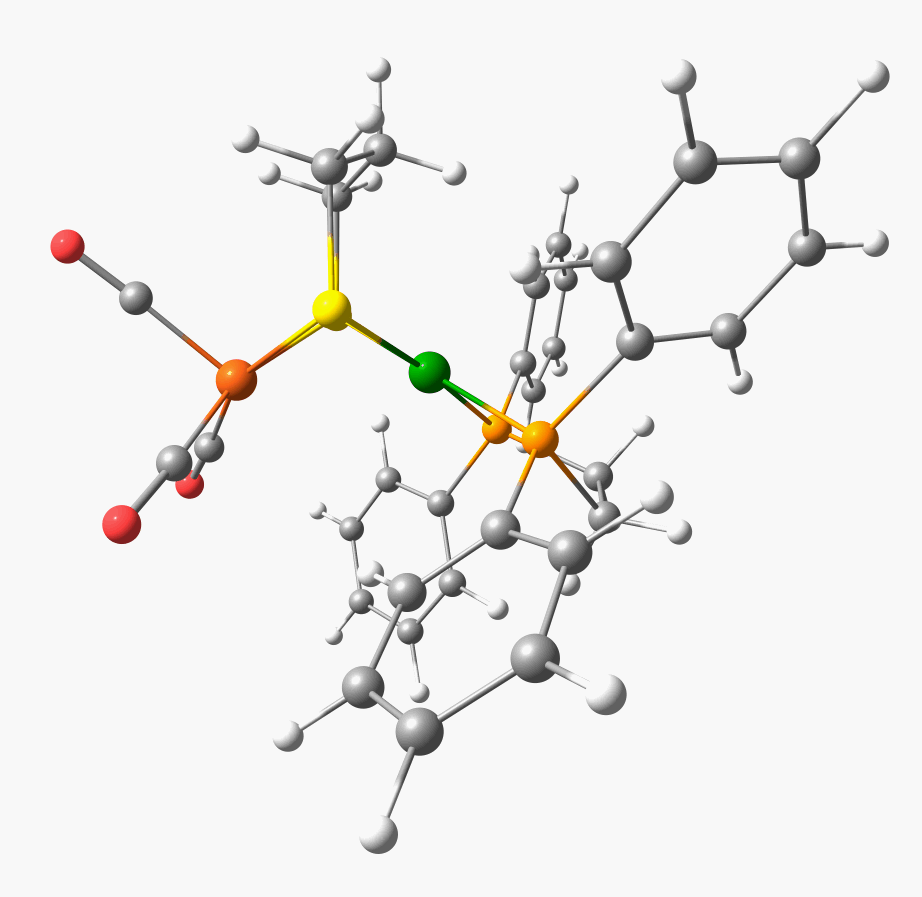

Supplement: Supplementary file 2 [file CC-050-C4CC04572F-s002.zip › DFT-calculated modes for 1' and [1']+/DFT-calculated modes for [1']+/1prime-plus_DFT_320cm-1.gif]

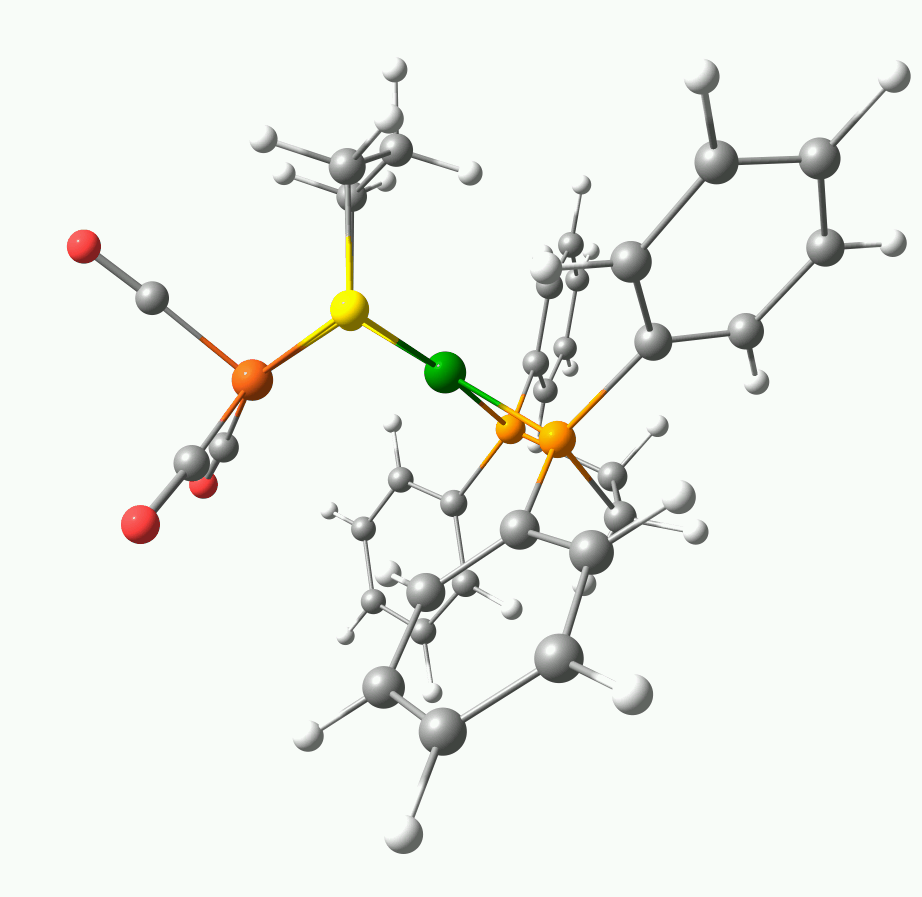

Supplement: Supplementary file 2 [file CC-050-C4CC04572F-s002.zip › DFT-calculated modes for 1' and [1']+/DFT-calculated modes for [1']+/1prime-plus_DFT_460cm-1.gif]

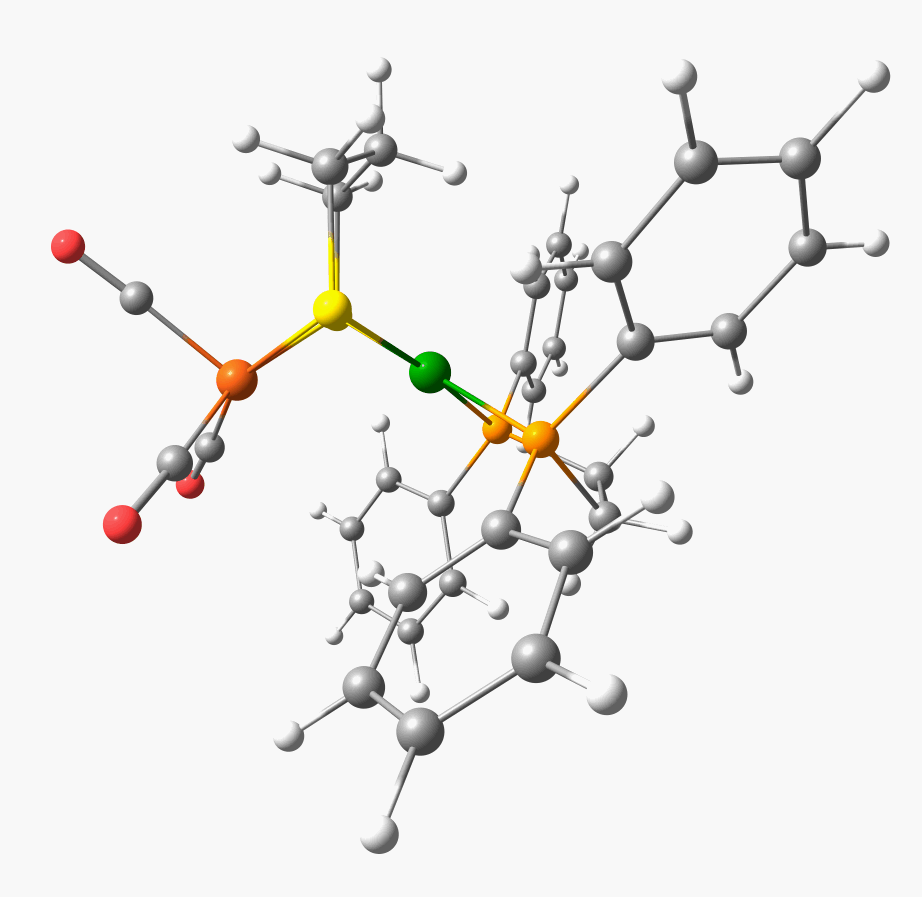

Supplement: Supplementary file 2 [file CC-050-C4CC04572F-s002.zip › DFT-calculated modes for 1' and [1']+/DFT-calculated modes for [1']+/1prime-plus_DFT_493cm-1.gif]

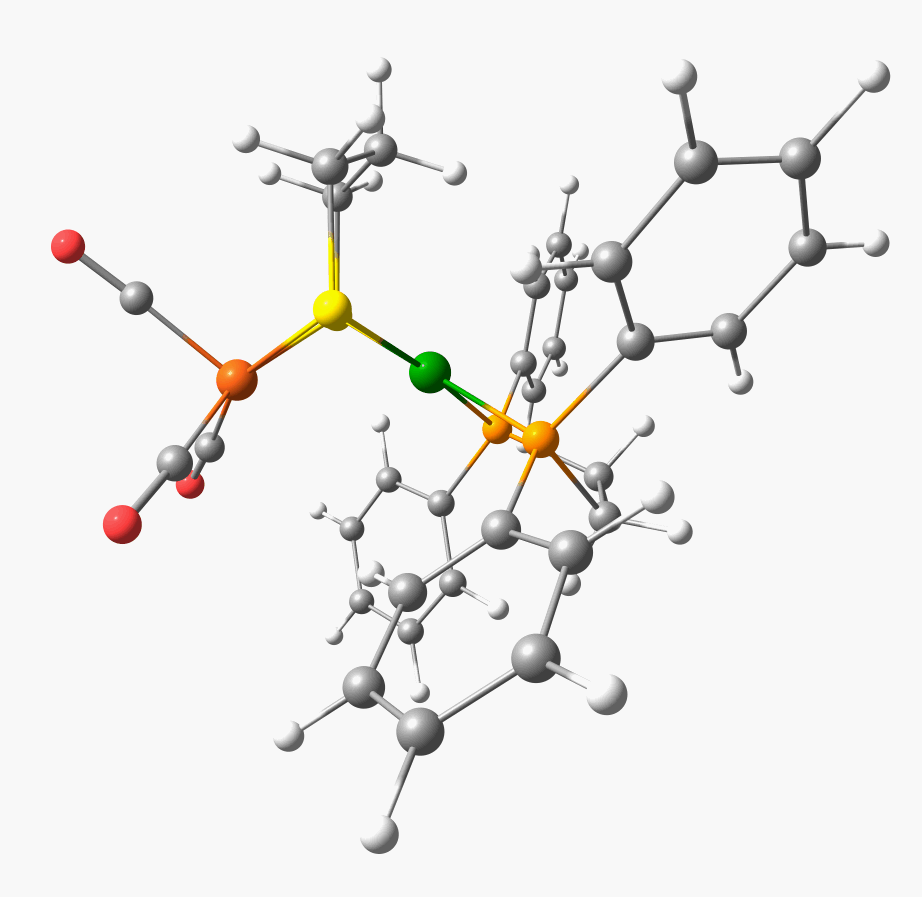

Supplement: Supplementary file 2 [file CC-050-C4CC04572F-s002.zip › DFT-calculated modes for 1' and [1']+/DFT-calculated modes for [1']+/1prime-plus_DFT_539cm-1.gif]

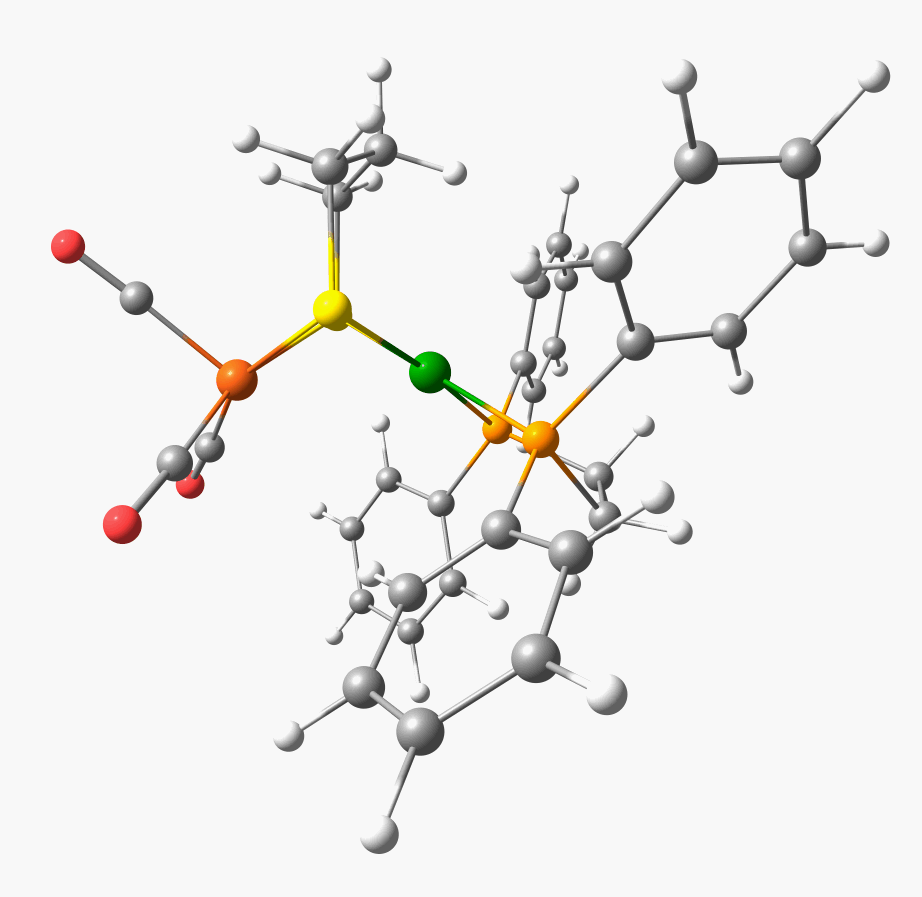

Supplement: Supplementary file 2 [file CC-050-C4CC04572F-s002.zip › DFT-calculated modes for 1' and [1']+/DFT-calculated modes for [1']+/1prime-plus_DFT_567cm-1.gif]

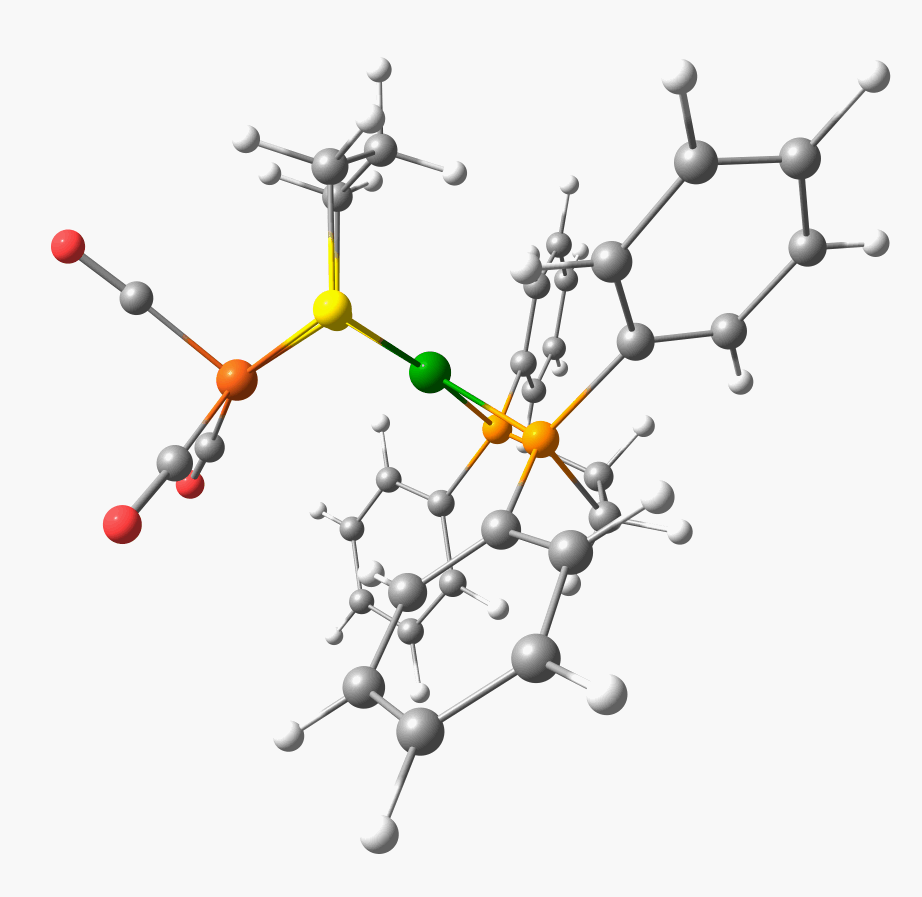

Supplement: Supplementary file 2 [file CC-050-C4CC04572F-s002.zip › DFT-calculated modes for 1' and [1']+/DFT-calculated modes for [1']+/1prime-plus_DFT_595cm-1.gif]
